# Gene Expression Patterns in Bone Following Mechanical Loading

**DOI:** 10.1002/jbmr.193

**Published:** 2010-07-23

**Authors:** Sara M Mantila Roosa, Yunlong Liu, Charles H Turner

**Affiliations:** 1 Department of Biomedical Engineering, Purdue UniversityWest Lafayette, IN, USA; 2 Division of Biostatistics, Department of Medicine, Center for Computational Biology and Bioinformatics, Indiana University School of MedicineIndianapolis, IN, USA; 3 Department Orthopaedic Surgery, Indiana University School of MedicineIndianapolis, IN, USA; 4 Department of Biomedical Engineering, Indiana University/Purdue UniversityIndianapolis, IN, USA

**Keywords:** EXON ARRAYS, GENE EXPRESSION, MECHANICAL LOADING

## Abstract

The advent of high-throughput measurements of gene expression and bioinformatics analysis methods offers new ways to study gene expression patterns. The primary goal of this study was to determine the time sequence for gene expression in a bone subjected to mechanical loading during key periods of the bone-formation process, including expression of matrix-related genes, the appearance of active osteoblasts, and bone desensitization. A standard model for bone loading was employed in which the right forelimb was loaded axially for 3 minutes per day, whereas the left forearm served as a nonloaded contralateral control. We evaluated loading-induced gene expression over a time course of 4 hours to 32 days after the first loading session. Six distinct time-dependent patterns of gene expression were identified over the time course and were categorized into three primary clusters: genes upregulated early in the time course, genes upregulated during matrix formation, and genes downregulated during matrix formation. Genes then were grouped based on function and/or signaling pathways. Many gene groups known to be important in loading-induced bone formation were identified within the clusters, including AP-1-related genes in the early-response cluster, matrix-related genes in the upregulated gene clusters, and Wnt/β-catenin signaling pathway inhibitors in the downregulated gene clusters. Several novel gene groups were identified as well, including chemokine-related genes, which were upregulated early but downregulated later in the time course; solute carrier genes, which were both upregulated and downregulated; and muscle-related genes, which were primarily downregulated. © 2011 American Society for Bone and Mineral Research.

## Introduction

Bone responds in an anabolic manner to physiologic dynamic loading. For example, the midshaft humerus in the throwing arm of baseball pitchers and catchers showed enhanced bone mass, structure, estimated strength, and resistance to torsion compared with the nonthrowing control arm.(1) In contrast, bone mineral density (BMD) in astronauts decreased 1.0% and 1.5% in the spine and hip, respectively, per month of spaceflight.(2,3)

Mechanical loading is a potent anabolic stimulus that substantially strengthens bones. Using the rat forelimb loading model, we showed that loading for only 3 minutes/day over 16 weeks doubled the energy necessary to break the ulna(4) and increased the fatigue life of the bone by 100-fold.(5) Mechanical loading uses pathways currently being investigated for new drug development, such as low density lipoprotein receptor–related protein 5 (LRP5) and sclerostin.(6) Others have proposed that the mechanical loading response is fundamental to bone biology, and many drugs work by modulating the responsiveness of bone to loading.(7) Hence, we believe that mechanical loading can be used as a paradigm for bone anabolism. This approach has the advantage of producing a local response, so the contralateral limb can be used as a control to compare with loading effects on the loaded limb.

The time course of bone formation after initiating mechanical loading is well characterized. New osteoblasts appear on the bone surface 24 to 48 hours after initiating mechanical loading,(8) and bone formation is observed within 96 hours of loading.(9) Bone formation increases between 5 and 12 days after starting loading,(9) but after 6 weeks of loading, bone formation returns to baseline levels.(10) These data indicate that applied mechanical loading to bone results in osteoblast recruitment followed by matrix production, which lasts for around 5 weeks before declining to baseline levels.(11) Mechanical loading affects signaling pathways and gene expression in loaded bone,(12,13) so we hypothesize that the gene expression patterns are time-dependent, with early activities stimulating a chain reaction of events that directly affects bone cell behavior and ultimately leads to bone formation.

The primary goal of this study was to determine the time sequence for gene expression in a bone subjected to mechanical loading. We evaluated loading-induced gene expression over a time course of 4 hours to 32 days. We then used bioinformatics tools to cluster genes into similar expression patterns and group genes within common signaling pathways.

## Materials and Methods

### Animals

Adult female Lewis rats were purchased from Charles River Laboratories, Inc. (Wilmington, MA, USA). The animals were fed standard rat chow and water *ad libitum* and acclimated until 20 weeks of age (average weight of 209.1 ± 12.5 g). Animals were divided into 11 groups: 4 hours (*n* = 9), 12 hours (*n* = 10), 1 day (*n* = 9), 2 days (*n* = 10), 4 days (*n* = 10), 6 days (*n* = 10), 8 days (*n* = 8), 12 days (*n* = 7), 16 days (*n* = 9), 24 days (*n* = 11), and 32 days (*n* = 12). All procedures were performed in accordance with the Institutional Animal Care and Use Committee guidelines of Indiana University.

### Mechanical loading

A standard model for bone loading was employed in which the right forelimb was loaded axially for 3 minutes per day while the left forearm served as a nonloaded contralateral control.(4,14,15) Prior to loading, animals were anesthetized with 3.0% isoflurane administered at a flow rate of 1.5 L/min. Compressive load was applied as an oscillating Haversine waveform for 360 cycles at a frequency of 2 Hz using a Bose ElectroForce 3200 Series electromechanical actuator (EnduraTEC, Eden Prairie, MN, USA). The peak load achieved during loading was 13 N, which has been shown previously to be anabolic.(14) Rats were subjected to loading sessions every day with 24 hours between sessions. The study groups listed earlier are referenced to the number of days (or hours) after the first bout of bone loading was applied. At the appropriate time point, animals were anesthetized with isoflurane and euthanized by cervical dislocation.

### Histology

Nine additional adult female Lewis rats were subjected to the loading protocol for histologic analysis. These rats were euthanized 1 and 4 days after beginning loading. The shafts of the right and left forearms with intact ulnae and radii were dissected, freed of excess muscle, and fixed in 10% neutral buffered formalin (NBF) for 48 hours. The fixed forearms were decalcified in a 70:30 solution of 10% ethylenediamine tetraacetic acid (EDTA) and 4% phosphate-buffered formalin (PBF) for 4 weeks. After decalcification, forearms were embedded in paraffin and sectioned at the ulnar midshaft at 4 µm. Sections were stained with hematoxylin and eosin (H&E) and used to identify active osteoblasts on the periosteal surfaces of loaded ulnae. Active osteoblasts were counted and defined as osteoblast cell bodies that were plump and present in the layer of cells immediately adjacent to newly formed osteoid on the bone surface. The sections were photographed on a Nikon Optiphot-2 microscope (Nikon, Inc., Melville, NY, USA) using 5× and 40× objectives and imported into Image-Pro Plus 6.3 (MediaCybernetics, Inc., Bethesda, MD, USA) analysis software for quantification. Total perimeter of the periosteal surface was measured, and average osteoblast number per total bone perimeter (mm, Ob.N/B.Pm) was reported.

### RNA isolation

The shafts of the right and left ulnae were dissected, freed of all soft tissue, and snap frozen in liquid nitrogen. The ulnae were stored at –80°C until RNA isolation. RNA was extracted using Trizol (Invitrogen, Carlsbad, CA, USA) and RNeasy Mini Kits (Qiagen, Inc., Valencia, CA, USA). Frozen ulnae were placed into a mortar containing liquid nitrogen and crushed with a pestle. The crushed bone was homogenized in Trizol, incubated, and centrifuged. The supernatant was removed, and RNeasy Mini Kits were used to isolate RNA. RNA was treated with a DNA-free kit (Ambion, Austin, TX, USA) to remove any residual DNA. RNA quality and quantity were determined using a spectrophotometer (NanoDrop, Wilmington, DE, USA). A paired *t* test was used to compare average total RNA quantity obtained from loaded and control bones for each time group. Average RNA quantity and standard errors were reported, and a *p* value < .05 was considered statistically significant.

### Quantitative polymerase chain reaction (qPCR)

Three matched RNA samples from loaded and control ulnae for each time group were used for quantitative real-time PCR (qPCR) experiments. RNA was reverse transcribed using the SuperScript III kit with oligo(dT) primers (Invitrogen). cDNA was diluted to a concentration of 2.5 ng/µL and used in qPCR reactions. A portion of the rat *collagen type 1α1* (*Col1a1*) gene sequence was amplified using a Taqman gene expression assay (assay ID: Rn01463848_m1, Applied Biosystems, Inc., Carlsbad, CA, USA). Serial dilutions of a single sample were amplified to calculate relative expression levels, which then were standardized to *β-actin* expression to facilitate comparison among samples. The reactions were performed on an ABI 7900HT Fast Real-Time PCR System (Applied Biosystems). A paired *t* test was used to compare *Col1a1* expression in loaded and control conditions. Average fold change and standard errors were reported, and *p* < .05 was considered statistically significant.

### Exon arrays

**Fig. 1:**
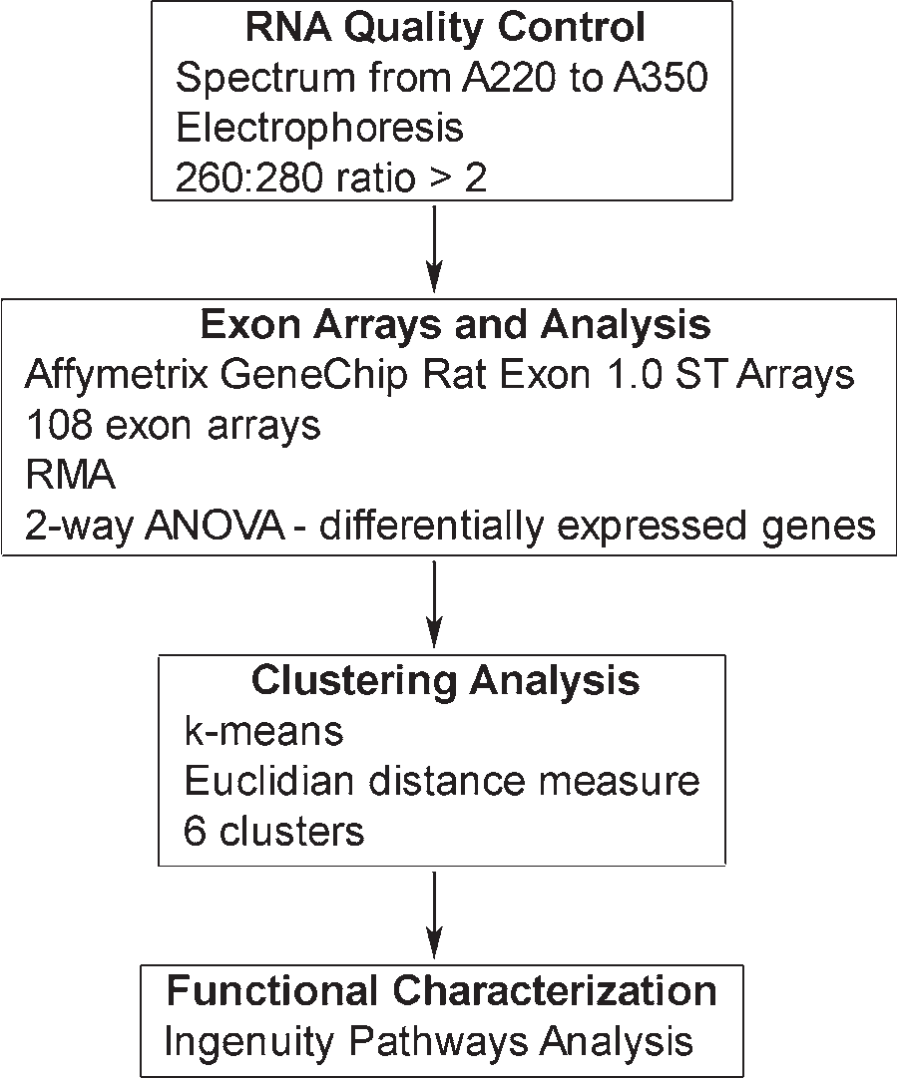
Flow diagram of the array analysis methods.

The array methodology is summarized in a flow diagram (Fig. 1). Quality-control measures were employed to ensure that high-quality RNA would be hybridized to exon arrays. A high-quality RNA sample was defined as having a minimum 260:280 ratio of 2.00. Four RNA samples had a 260:280 ratio of slightly less than 2.00, and these samples were chosen to optimize the quantity of total RNA as well as the quality. The range of 260:280 ratios of all samples used was 1.96 to 2.31.

Five matched (control and loaded) ulna RNA samples from each time group were used for exon array analysis. One exception to this was the 12-day group, where only four matched samples were used. RNA from the control and loaded ulnae from 54 animals were analyzed on separate arrays. The exon array hybridizations were carried out using the facilities of the Center for Medical Genomics (CMG) at Indiana University School of Medicine. One microgram of each sample was labeled and hybridized using the Affymetrix WT protocol [GeneChip Whole Transcript (WT) Sense Target Labeling Assay Manual, Version 4, Affymetrix, Santa Clara, CA, USA]. All processing was done in balanced batches. The exon arrays were scanned using the GeneChip Scanner 3000 using the Affymetrix GeneChip Operating System (GCOS). Data were exported for analysis in the Partek Genomics Suite (Partek, Inc., St. Louis, MO, USA).

The robust multiarray average (RMA) algorithm(16–18) was used to import raw data from the core probe sets, which represented over 8000 genes. A two-way ANOVA model [variables were loading condition (loaded or control) and animal] was used to identify differentially expressed genes, which were defined as having *p* < .01 and fold change beyond ±1.4. Probe sets with a signal value of less than 3 were not reliable and were excluded from analysis. Raw data and analyzed data were MIAME-compliant(19) and were deposited in the Gene Expression Omnibus database (series accession number GSE22286).

### Clustering analysis

Differentially expressed genes were further analyzed with a *k*-means (ie, partitioning) clustering algorithm. The inclusion criterion for a gene to qualify for clustering analysis was that the gene must be differentially expressed at a minimum of one of the 11 time points.

### Functional characterization

Groups of genes then were defined based on gene and associated protein function using Ingenuity Pathways Analysis (IPA, Ingenuity Systems, Redwood City, CA, USA, www.ingenuity.com). IPA uses information about gene relationships from the literature to characterize gene sets, create gene networks, and identify important signaling pathways in gene expression data.

## Results

### Histology

**Fig. 2:**
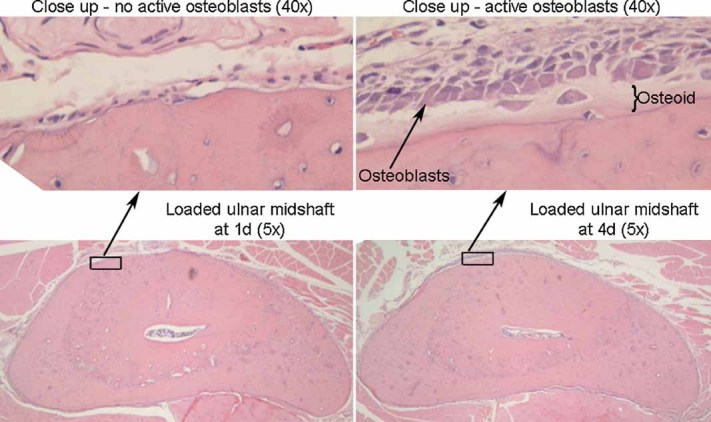
Active osteoblasts were observed on the periosteal surface in H&E-stained cross sections of the loaded ulnar midshaft at 4 days. (*Bottom*) Cross sections of representative loaded ulnae at 1 day (*left*) and 4 days (*right*) (×5). (*Top*) Close-up images of the periosteal surface on the medial side of loaded ulnae at 1 day (*left*) and 4 days (*right*) (×40). Many active osteoblasts and osteoid were present on the bone surface at 4 days, which indicated that new bone was being formed.

H&E-stained sections through the ulnar midshaft were used to count osteoblasts and look for evidence of bone formation (Fig. 2). There were no osteoblasts on the periosteal surface of control ulnae at either 1 or 4 days after loading. Some osteoblasts were present on the periosteal surface of a single loaded bone on 1 day, but no active osteoblasts were observed in any other loaded bones on 1 day. The average Ob.N/B.Pm was 2.22 ± 4.97 for the loaded bones 1 day after loading. The most remarkable results were in loaded bones in the 4-day group. Osteoid was observed on the periosteal surface of loaded bones, which indicated that new bone was being formed. In addition, active osteoblasts were present and the average Ob.N/B.Pm was 33.1 ± 6.6 for loaded bones at 4 days. Osteoclasts were not observed in any of the bone sections.

### Total RNA

**Fig. 3:**
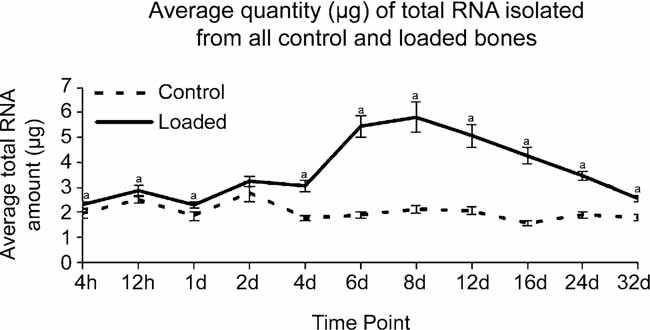
The amount of total RNA isolated from loaded bones was significantly greater than the amount of total RNA isolated from control bones at all time points except 2 days. A paired *t* test was used to compare RNA quantities in loaded and control ulnae from matched RNA samples (^a^*p* value < .05). Standard errors are indicated.

The total amount of RNA isolated from loaded bones was significantly greater than the total amount of RNA isolated from control bones for all time points except 2 days (Fig. 3). Total RNA increased substantially from 4 to 8 days in the loaded bones, and then the amount declined from 12 to 32 days.

### *Col1a1* expression

**Fig. 4:**
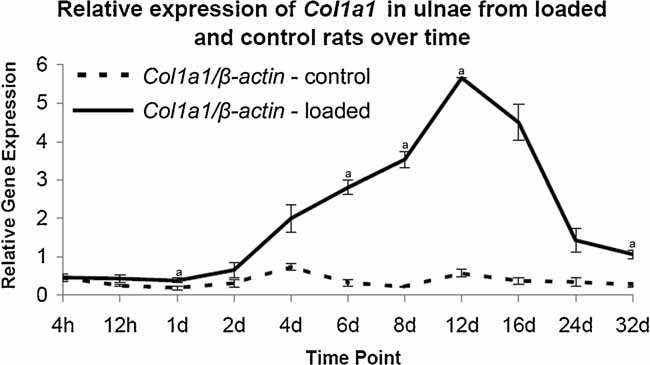
*Col1a1* expression increased in loaded ulnae at 1, 6, 8, 12, and 32 days. qPCR was used to evaluate *Col1a1* gene expression in loaded and control ulnae across the time course. *Col1a1* expression was normalized to *β-actin* expression to facilitate comparison among samples. A paired *t* test was used to compare expression in loaded and control conditions (^a^*p* < .05). Standard errors are indicated.

qPCR was used to measure *Col1a1*, a major bone matrix gene, to characterize matrix synthesis.(20–22) Figure 4 shows that *Col1a1* gene expression began to increase in loaded ulnae at 4 days, when active osteoblasts were present on the periosteal bone surface (Fig. 2). *Col1a1* expression peaked at 12 days in loaded bones and declined toward baseline levels at later time points. *Col1a1* expression was not changed in control ulnae. Since *Col1a1* expression corresponded with the observation of active osteoblasts, we presume that osteoblasts were the primary contributors to the qPCR signal for *Col1a1*. Furthermore, the *Col1a1* expression time course was very similar to the time course of osteoblast recruitment and bone formation observed by others.(8–10)

### Clustering analysis

**Fig. 5:**
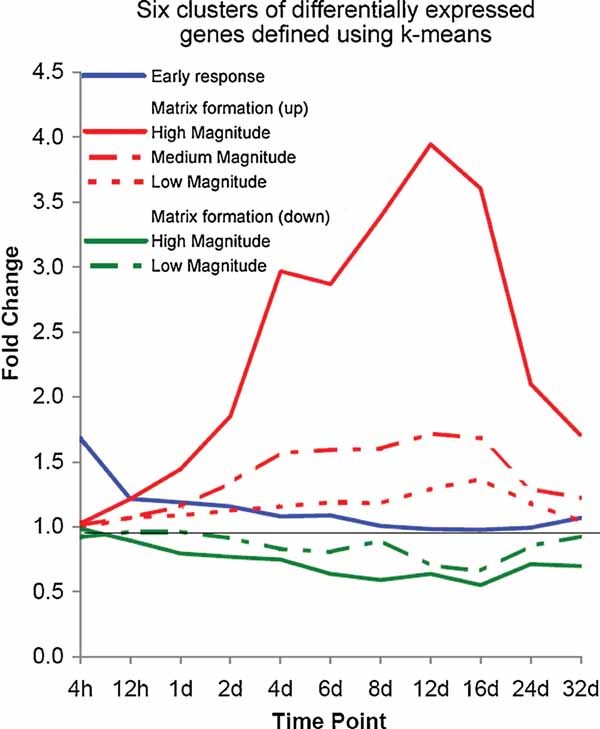
Six clusters were defined using a *k*-means clustering algorithm. Genes in the early response cluster (*n* = 88) were upregulated primarily at 4 hours. Expression of genes in the matrix-formation (up) clusters peaked between 12 and 16 days, and this cluster was subdivided into three magnitudes: high (*n* = 23), medium (*n* = 182), and low (*n* = 308). Expression of genes in the matrix-formation (down) clusters peaked at 16 days, and this cluster was subdivided into two magnitudes: high (*n* = 124) and low (*n* = 326).

The primary goal of the clustering analysis was to determine the patterns of gene expression in bones subjected to mechanical loading over a time sequence of 4 hours to 32 days. We identified 1051 genes that were differentially expressed at a minimum of one of the 11 time points. This gene list was analyzed using *k*-means clustering. Six distinct patterns of differential gene expression were identified over the time course (Fig. 5): an early-response cluster (blue line) in which the genes were upregulated early but not late in the time course, three matrix-formation (up) clusters (red lines) that followed the pattern of matrix synthesis illustrated by expression of the *Col1a1* gene (Fig. 4), and two matrix-formation (down) clusters (green lines) that were downregulated during matrix formation. There were 88 genes in the early-response cluster, and genes in this cluster were upregulated early on, primarily at 4 hours. There were 23 genes in the matrix-formation (up) high-magnitude cluster, 182 genes in the matrix-formation (up) medium-magnitude cluster, and 308 genes in the matrix-formation (up) low-magnitude cluster. Genes in these three clusters were upregulated during matrix formation, and expression reached peak levels between 12 and 16 days. There were 124 genes in the matrix-formation (down) high-magnitude cluster and 326 genes in the matrix-formation (down) low-magnitude cluster. Genes in these two clusters were downregulated during matrix formation, and differential expression was greatest at 16 days. Within each cluster of genes, we identified groups of genes that have common functions or are part of common pathways using IPA. The complete list of 1051 genes is available in Supplemental Table S1.

### Early-response cluster

Selected gene groups from the early-response cluster are listed in Table 1 and included AP-1, calcium signaling, chemokine, cytokine, and matrix. Of these, the chemokine group that was upregulated within 12 hours of loading was novel for bone formation. Genes in the chemokine group included a binding protein (*Ccbp2*), C–C motif ligands (*Ccl2* and *Ccl7*), and C–X–C motif ligands (*Cxcl1* and *Cxcl13*).

**Table 1:** Selected Gene Groups Present in the Early-Response Cluster, Which Were Upregulated 4 Hours After a Single Loading Session

Gene group	Gene name
AP-1	
*Fosl1*	Fos-like antigen 1
*Junb*	Jun B protooncogene
Calcium signaling	
*Anxa2*	Annexin A2
*S100a4*	S100 calcium-binding protein A4
*S100a10*	S100 calcium-binding protein A10
Chemokine	
*Ccbp2*	Chemokine-binding protein 2
*Ccl2*	Chemokine (C–C motif) ligand 2
*Ccl7*	Chemokine (C–C motif) ligand 7
*Cxcl1*	Chemokine (C–X–C motif) ligand 1
*Cxcl13*	Chemokine (C–X–C motif) ligand 13
Cytokine	
*Il1rl1*	Interleukin 1 receptor–like 1
*Il1rl2*	Interleukin 1 receptor–like 2
*Osm*	Oncostatin M
*Osmr*	Oncostatin M receptor
*Socs3*	Suppressor of cytokine signaling 3
*Stat3*	Signal transducer and activator of transcription 3
*Tnfrsf12a*	Tumor necrosis factor receptor superfamily, member 12A
Matrix	
*Adamts1*	ADAM metallopeptidase with thrombospondin type 1 motif
*Ecm1*	Extracellular matrix protein 1
*Serpina3n*	Serine (or cysteine) peptidase inhibitor, clade A, member 3N
*Serpine1*	Serpin peptidase inhibitor, clade E, member 1
*Tfpi2*	Tissue factor pathway inhibitor 2
Other	
*Ccnd2*	Cyclin d2
*Clic1*	Chloride intracellular channel 1
*Gpr1*	G protein–coupled receptor 1
*Kcne4*	Potassium voltage-gated channel, Isk-related subfamily, gene 4
*Lep*	Leptin
*Sdc4*	Syndecan 4

### Matrix-formation (up) clusters

Expression of genes in the matrix-formation (up) clusters peaked during the synthetic phase of bone formation. Several important gene groups were identified and include apoptosis, calcium signaling, cytokine, growth factor, ion channel, matrix, muscle, neurotransmitter, solute carrier, transforming growth factor β (TGF-β) signaling, and Wnt/β-catenin signaling (Table 2). The matrix group included many of the expected genes associated with bone formation, such as *Alpl*, *Bglap*, and *Col1a2*. Probes targeting the *Col1a1* gene were not detected on the exon array and therefore *Col1a1* is not reported in Table 2, but using qPCR, we found that its expression followed a similar pattern (Fig. 4). The solute carrier group represents a novel finding with respect to bone mechanotransduction. This group includes transporters for amino acids and many ions. Presumably, such transport is necessary to facilitate the synthetic activity of osteoblasts.

**Table 2:** Selected Gene Groups Present in the Bone-Formation (Up) Clusters, Which Were Upregulated During Matrix Formation

Gene group	Cluster	Gene name
Apoptosis		
*Aifm3*	L	Apoptosis-inducing factor, mitochondrion-associated 3
*Bok*	L	BCL2-related ovarian killer
*Dad1*	L	Defender against cell death 1
Calcium signaling		
*Atp2b2*	L	ATPase, Ca^2+^ transporting, plasma membrane 2
*Cacnb3*	M	Calcium channel, voltage-dependent, β3 subunit
*Cadps*	M	Ca^2+^-dependent secretion activator
*Calu*	M	Calumenin
*Capn6*	M	Calpain 6
*Flvcr2*	L	Feline leukemia virus subgroup C cellular receptor family, member 2
*Hpcal1*	L	Hippocalcin-like 1
*Stc1*	L	Stanniocalcin 1
*Syt3*	L	Synaptotagmin III
Cytokine		
*Il4*	L	Interleukin 4
*Il11*	L	Interleukin 11
*Il12a*	M	Interleukin 12a
*Socs2*	L	Suppressor of cytokine signaling 2
Growth factor		
*Fgf14*	L	Fibroblast growth factor 14
*Pdgfa*	L	Platelet-derived growth factor α polypeptide
*Pdgfc*	M	Platelet-derived growth factor C
*Pdgfrl*	M	Platelet-derived growth factor receptor–like
*Pgf*	L	Placental growth factor
Ion channel		
*Kcne3*	M	Potassium voltage-gated channel, Isk-related subfamily, gene 3
*Kcng3*	L	Potassium voltage-gated channel, subfamily G, member 3
*Kcnk6*	M	Potassium inwardly rectifying channel, subfamily K, member 6
*Kcnma1*	M	Potassium large conductance calcium-activated channel, subfamily M, α member 1
*Kcnn2*	M	Potassium intermediate/small conductance calcium-activated channel, subfamily N, member 2
*Kcns1*	L	Potassium voltage-gated channel, delayed-rectifier, subfamily S, member 1
*Scn1a*	L	Sodium channel, voltage-gated, type I, α
Matrix		
*Acan*	H	Aggrecan
*Alpl*	M	Alkaline phosphatase, liver/bone/kidney
*Bglap*	M	Bone γ-carboxyglutamate (gla) protein (osteocalcin)
*Bgn*	M	Biglycan
*Col1a2*	M	Collagen, type I, α2
*Col2a1*	M	Collagen, type II, α1
*Col3a1*	M	Collagen, type III, α1
*Col5a1*	H	Collagen, type V, α1
*Col11a2*	H	Collagen, type XI, α2
*Col16a1*	M	Collagen, type XVI, α1
*Cthrc1*	H	Collagen triple-helix-repeat-containing 1
*Efemp2*	L	EGF-containing fibulin-like extracellular matrix protein 2
*Fn1*	M	Fibronectin 1
*Ibsp*	M	Integrin-binding sialoprotein
*Lepre1*	H	Leucine proline–enriched proteoglycan (leprecan) 1
*Lox*	H	Lysyl oxidase
*Mmp2*	M	Matrix metallopeptidase 2
*Mmp9*	M	Matrix metallopeptidase 9
*Mmp14*	M	Matrix metallopeptidase 14
*Mmp16*	M	Matrix metallopeptidase 16
*Mmp23*	M	Matrix metallopeptidase 23
*Omd*	M	Osteomodulin
*P4ha1*	M	Procollagen-proline, 2-oxoglutarate 4-dioxygenase (proline 4-hydroxylase), α polypeptide I
*P4ha3*	M	Procollagen-proline, 2-oxoglutarate 4-dioxygenase (proline 4-hydroxylase), α polypeptide III
*P4hb*	M	Prolyl 4-hydroxylase, β polypeptide
*Pcolce*	M	Procollagen C-endopeptidase enhancer
*Plod2*	M	Procollagen lysine, 2-oxoglutarate 5-dioxygenase 2
*Plod3*	M	Procollagen-lysine, 2-oxoglutarate 5-dioxygenase 3
*Sparc*	L	Secreted protein, acidic, cysteine-rich (osteonectin)
*Timp1*	H	TIMP metallopeptidase inhibitor 1
*Vcan*	H	Versican
Muscle		
*Myo1b*	M	Myosin Ib
*Myo5a*	M	Myosin Va
*Tpm4*	L	Tropomyosin 4
*Tuba3a*	L	Tubulin, α3A
Neurotransmitter		
*Drd1a*	L	Dopamine receptor D1A
*Glrb*	L	Glycine receptor, β
*Htr1d*	L	5-Hydroxytryptamine (serotonin) receptor 1D
*Htr2a*	M	5-hydroxytryptamine (serotonin) receptor 2A
*Syn1*	L	synapsin I
Solute Carrier		
*Slc1a4*	L	solute carrier family 1 (glutamate/neutral amino acid transporter), member 4
*Slc2a13*	M	solute carrier family 2 (facilitated glucose transporter), member 13
*Slc5a5*	L	solute carrier family 5 (sodium iodide symporter), member 5
*Slc6a2*	M	Solute carrier family 6 (neurotransmitter transporter, noradrenalin), member 2
*Slc6a15*	M	Solute carrier family 6 (neutral amino acid transporter), member 15
*Slc7a8*	L	Solute carrier family 7 (cationic amino acid transporter, y+ system), member 8
*Slc8a3*	M	Solute carrier family 8 (sodium/calcium exchanger), member 3
*Slc9a2*	H	Solute carrier family 9 (sodium/hydrogen exchanger), member 2
*Slc9a4*	M	Solute carrier family 9 (sodium/hydrogen exchanger), member 4
*Slc13a5*	H	Solute carrier family 13 (sodium-dependent citrate transporter), member 5
*Slc25a1*	L	Solute carrier family 25 (mitochondrial carrier, citrate transporter), member 1
*Slc26a2*	L	Solute carrier family 26 (sulfate transporter), member 2
*Slc30a4*	M	Solute carrier family 30 (zinc transporter), member 4
*Slc31a1*	M	Solute carrier family 31 (copper transporters), member 1
*Slc35b1*	L	Solute carrier family 35, member B1
*Slc36a1*	M	Solute carrier family 36 (proton/amino acid symporter), member 1
*Slc36a2*	H	Solute carrier family 36 (proton/amino acid symporter), member 2
*Slc39a7*	M	Solute carrier family 39 (zinc transporter), member 7
*Slc39a8*	M	Solute carrier family 39 (metal ion transporter), member 8
*Slco3a1*	L	Solute carrier organic anion transporter family, member 3a1
TGF-β signaling		
*Bambi*	M	BMP and activin membrane–bound inhibitor
*Bmp2*	L	Bone morphogenetic protein 2
*Hnf4a*	L	Hepatocyte nuclear factor 4, α
*Inhba*	L	Inhibin β-A
*Nkx2-5*	L	NK2 transcription factor related, locus 5
*Smad9*	M	SMAD family member 9
*Tgfb1*	L	Transforming growth factor, β1
*Tgfb3*	L	Transforming growth factor, β3
*Vdr*	L	Vitamin D (1,25-dihydroxyvitamin D3) receptor
Wnt/β-catenin signaling		
*Cdh2*	M	Cadherin 2
*Cpz*	H	Carboxypeptidase Z
*Gja1*	M	Gap junction protein, α1
*Kremen1*	M	Kringle-containing transmembrane protein 1
*Wif1*	M	Wnt inhibitory factor 1
*Wisp1*	M	Wnt1 inducible signaling pathway protein 1
Other		
*Ccnd1*	M	Cyclin D1
*Creb3l1*	H	cAMP-responsive element–binding protein 3-like 1
*Foxs1*	L	Forkhead box S1
*Hif1a*	L	Hypoxia-inducible factor 1, α subunit
*Jund*	L	Jun D protooncogene
*Pthr1*	M	Parathyroid hormone receptor 1
*Sox11*	L	SRY (sex-determining region Y)-box 11
*Sp7*	M	Sp7 transcription factor (osterix)

H = high-magnitude cluster; M = medium-magnitude cluster; L = low-magnitude cluster.

### Matrix-formation (down) clusters

Genes in the matrix-formation (down) clusters were downregulated during the synthetic phase of bone formation. Several important gene groups were identified in these clusters and include calcium signaling, cell cycle, chemokine, cytokine, growth factor, ion channel, matrix, neurotransmitter, TGF-β signaling, ubiquitin, and inhibitors of Wnt/β-catenin signaling (Table 3). In particular, there were large groups of muscle and solute carrier genes that were downregulated. Another important finding in this cluster was that genes encoding inhibitors of both TGF-β (*Grem1* and *Chrdl1*) and Wnt/β-catenin signaling (*Sost* and *Sfrp4*) were downregulated, supporting observations that negative regulation of inhibitors was important to enhance signaling through these pathways.(21)

**Table 3:** Selected Gene Groups Present in the Bone-Formation (Down) Clusters, Which Were Downregulated During Matrix Formation

Gene group	Cluster	Gene name
Calcium signaling		
*Asph*	H	Aspartate-β-hydroxylase
*Cab39l*	L	Calcium-binding protein 39-like
*Calb2*	H	Calbindin 2
*Camk2b*	H	Calcium/calmodulin-dependent protein kinase (CaM kinase) IIβ
*Casq2*	H	Calsequestrin 2
*Pvalb*	H	Parvalbumin
*Stc2*	L	Stanniocalcin 2
*Trdn*	H	Triadin
Cell cycle		
*Ccna2*	L	Cyclin A2
*Ccnb1*	L	Cyclin B1
*Cdc25b*	L	Cell division cycle 25 homologue B
*Cenpc1*	L	Centromere protein C1
Chemokine		
*Ccl11*	L	Chemokine (C–C motif) ligand 11
*Ccr1*	L	Chemokine (C–C motif) receptor 1
*Ccr2*	L	Chemokine (C–C motif) receptor 2
*Ccr3*	L	Chemokine (C–C motif) receptor 3
*Cxcl12*	L	Chemokine (C–X–C motif) ligand 12
*Cxcl14*	H	Chemokine (C–X–C motif) ligand 14
*Cxcr5*	L	Chemokine (C–X–C motif) receptor 5
Cytokine		
*Asb2*	H	Ankyrin repeat and SOCS box-containing 2
*Il8rb*	L	Interleukin 8 receptor, β
*Tnfsf10*	H	Tumor necrosis factor (ligand) superfamily, member 10 (TRAIL)
*Tnfrsf14*	L	Tumor necrosis factor receptor superfamily, member 14
Growth factor		
*Egf*	L	Epidermal growth factor
*Fgf1*	L	Fibroblast growth factor 1
*Fgf7*	H	Fibroblast growth factor 7
*Fgf23*	H	Fibroblast growth factor 23
*Fgl2*	H	Fibrinogen-like 2
*Hgf*	L	Hepatocyte growth factor
*Igfbp6*	L	Insulin-like growth factor–binding protein 6
Ion channel		
*Cacnb1*	H	Calcium channel, voltage-dependent, β1 subunit
*Cacng1*	H	Calcium channel, voltage-dependent, γ subunit 1
*Cacng7*	H	Calcium channel, voltage-dependent, γ subunit 7
*Clca2*	L	Chloride channel calcium activated 2
*Clic5*	L	Chloride intracellular channel 5
*Hcn4*	L	Hyperpolarization activated cyclic nucleotide-gated potassium channel 4
*Kcnj3*	L	Potassium inwardly rectifying channel, subfamily J, member 3
*Kcnj11*	H	Potassium inwardly rectifying channel, subfamily J, member 11
*Kcnj12*	H	Potassium inwardly rectifying channel, subfamily J, member 12
*Kcnn4*	L	Potassium intermediate/small conductance calcium-activated channel, subfamily N, member 4
*Sclt1*	L	Sodium channel and clathrin linker 1
*Scn4a*	H	Sodium channel, voltage-gated, type 4, α subunit
*Scn4b*	H	Sodium channel, type IV, β
*Scn7a*	L	Sodium channel, voltage-gated, type VII, α
Matrix		
*Efemp1*	H	EGF-containing fibulin-like extracellular matrix protein 1
*Mmp8*	L	Matrix metallopeptidase 8
*Prelp*	L	Proline arginine–rich end leucine-rich repeat protein
*Serpinb2*	L	Serine (or cysteine) peptidase inhibitor, clade B, member 2
*Spon1*	L	Spondin 1
Muscle		
*Acta1*	H	Actin, α1
*Actn3*	H	Actinin, α3
*Ampd1*	H	Adenosine monophosphate deaminase 1 (isoform M)
*Des*	H	Desmin
*Dmd*	H	Dystrophin, muscular dystrophy
*Gyg1*	H	Glycogenin 1
*Mb*	H	Myoglobin
*Myl1*	H	Myosin, light polypeptide 1
*Mylk2*	H	Myosin light chain kinase 2
*Mylpf*	H	Myosin light chain, phosphorylatable, fast
*Myocd*	H	Myocardin
*Smpx*	H	Small muscle protein, X-linked
*Synm*	H	Synemin
*Tnni2*	H	Troponin I type 2
*Tnnt3*	H	Troponin T type 3
*Tpm2*	L	Tropomyosin 2
Neurotransmitter		
*Chrna1*	H	Cholinergic receptor, nicotinic, α1
*Chrnb1*	H	Cholinergic receptor, nicotinic, β1
*Chrne*	H	Cholinergic receptor, nicotinic, ɛ
*Htr1b*	L	5-Hydroxytryptamine (serotonin) receptor 1B
*Maob*	L	Monoamine oxidase B
Solute carrier		
*Slc2a1*	H	Solute carrier family 2 (facilitated glucose transporter), member 1
*Slc4a1*	L	Solute carrier family 4 (anion exchanger), member 1
*Slc6a4*	L	Solute carrier family 6 (neurotransmitter transporter, serotonin), member 4
*Slc6a20*	L	Solute carrier family 6 (neurotransmitter transporter), member 20
*Slc14a1*	L	Solute carrier family 14 (urea transporter), member 1
*Slc15a2*	L	Solute carrier family 15 (H^+^/peptide transporter), member 2
*Slc16a1*	L	Solute carrier family 16, member 1 (monocarboxylic acid transporter 1)
*Slc16a3*	H	Solute carrier family 16, member 3 (monocarboxylic acid transporter 4)
*Slc16a6*	L	Solute carrier family 16, member 6 (monocarboxylic acid transporter 7)
*Slc22a3*	H	Solute carrier family 22 (extraneuronal monoamine transporter), member 3
*Slc25a11*	L	Solute carrier family 25 (mitochondrial carrier, oxoglutarate carrier), member 11
*Slc25a30*	L	Solute carrier family 25, member 30
*Slc38a1*	L	Solute carrier family 38, member 1
*Slc38a3*	L	Solute carrier family 38, member 3
*Slco1a4*	H	Solute carrier organic anion transporter family, member 1a4
TGF-β signaling		
*Bmpr1b*	H	Bone morphogenetic protein receptor, type IB
*Chrdl1*	L	Chordin-like 1
*Grem1*	H	Gremlin 1, cysteine knot superfamily
*Tgfbr3*	L	Transforming growth factor, β receptor III
Ubiquitin		
*Usp1*	L	Ubiquitin-specific peptidase 1
*Usp15*	L	Ubiquitin-specific peptidase 15
Wnt/β-catenin signaling (inhibitors)		
*Sfrp4*	H	Secreted frizzled-related protein 4
*Sost*	L	Sclerosteosis
Other		
*Adrb2*	H	Adrenergic, β2, receptor, surface
*Casp1*	L	Caspase 1
*Fcn1*	L	Ficolin (collagen/fibrinogen domain containing) 1
*Lepr*	L	Leptin receptor
*Mustn1*	L	Musculoskeletal, embryonic nuclear protein 1
*Ptgds2*	L	Prostaglandin D2 synthase 2, hematopoietic
*Rsad2*	L	Radical *S*-adenosyl methionine domain containing 2

H = high-magnitude cluster; L = low-magnitude cluster.

## Discussion

We have identified six distinct patterns of gene expression, and these were categorized into three primary clusters of genes: early-response, matrix-formation (up), and matrix-formation (down). Genes within these clusters were grouped based on function and/or signaling pathways using IPA. Many gene groups known to be important in loading-induced bone formation were identified, as were several novel gene groups whose function with respect to bone formation is not known.

### Early-response cluster

The early-response cluster was characterized primarily by genes that showed increased expression 4 hours after a single loading session. It is known that gene expression of components in the AP-1 transcription factor complex increases with mechanical loading. *Fosl1* and *Junb* genes were both upregulated shortly after loading in our study and those of others,(23,24) and *Fosb* gene expression increased after loading and may be associated with osteoblast differentiation and bone formation.(25) Others have observed increased protein expression of FOS and JUN family members after loading in association with osteoblast proliferation.(26) JUND is one of the primary AP-1 signals when osteoblasts are fully differentiated during matrix production and mineralization, and JUND inhibits the S phase of the cell cycle.(26) Our results are consistent with these findings because *JunD* gene expression increased during the matrix-formation phase, which corresponded to the observation of differentiated osteoblasts on the bone surface.

A group of chemokines was identified in the early-response cluster and presents a potentially novel finding. Chemokines act as chemotactic molecules for a number of different cell types. Chemokines are known to be important in many biologic functions, including development, immune function, and wound repair.(27) Many cytokines and chemokines that function in the immune system regulate osteoblasts and osteoclasts,(28) and osteoblasts are known to produce both chemokines and their receptors.(29) The chemokine-related genes identified in our study included a binding protein (*Ccbp2*), C–C motif ligands (*Ccl2* and *Ccl7*), and C–X–C motif ligands (*Cxcl1* and *Cxcl13*). CXCL13 upregulates alkaline phosphatase activity and also induces release of β-*N*-acetylhexosaminidase, which is involved in bone remodeling.(30) There also was a group of chemokine-related genes that were downregulated during matrix formation, which included a C–C motif ligand (*Ccl11*), C–C motif receptors (*Ccr1*, *Ccr2*, and *Ccr3*), a C–X–C motif ligand (*Cxcl12*), and a C–X–C motif receptor (*Cxcr5*). Ligands that bind CCR1 are chemoattractants that promote recruitment of marrow cells that can develop into osteoclasts.(31) CCR2 is expressed on osteoclasts,(32) and CCR2 knockout mice have high bone mass, impaired osteoclast function, and are resistant to ovariectomy-induced bone loss.(33) The receptor for CXCL13, CXCR5, is expressed in osteoblasts.(30) We postulate that chemokines in the early-response cluster may be active shortly after a mechanical loading event to recruit osteoblasts and/or osteoblast precursors to a site of bone formation. However, later in the synthetic phase, additional osteoblasts are not necessary, so chemokines may be downregulated. It is also possible that differential expression of chemokines is related to the interaction of the bone cells and immune cells. CXCL12 plays a role in fetal bone marrow colonization,(34,35) and interaction of CXCL12 and its receptor, CXCR4, is necessary for retention of hematopoietic stem cells (HSCs) in adult bone marrow.(36,37) CXCL12/CXCR4 signaling modulates bone resorption,(38) osteoblast proliferation,(39) and priming of hematopoietic progenitor cells.(40,41) In addition, CXCL13/CXCR5 signaling is known to affect several aspects of B-cell functionality.(42,43)

A group of cytokines was present in the early-response cluster as well. In bone, cytokines are usually associated with osteoclasts and increased bone resorption. However, osteoclasts were not observed in any of the histologic sections we analyzed. Oncostatin M (OSM) is a cytokine that was shown recently to inhibit *Sost* gene expression in primary calvarial osteoblasts, and OSM treatment of calvariae in vivo enhanced bone formation.(44) In our study, *Osm* expression was highest at 4 hours and could have contributed to decreased *Sost* expression during the synthetic phase.

### Matrix-formation (up) and matrix-formation (down) clusters

Most genes exhibited differential expression during the synthetic phase of bone formation and belonged to the matrix-formation (up) and matrix-formation (down) clusters. The matrix-formation (up) clusters were comprised of three groups that exhibited different degrees of upregulation in response to loading, and the matrix-formation (down) clusters were comprised of two groups that were downregulated in response to loading. Matrix genes dominated the upregulated clusters, whereas muscle-related genes dominated the downregulated clusters. Several gene groups were expressed in both up- and downregulated clusters, including solute carriers, Wnt/β-catenin signaling, and TGF-β signaling.

The largest group of upregulated genes identified in the synthetic phase was associated with extracellular matrix and had 31 members, including proteoglycans, collagens, genes related to collagen synthesis, osteoblast markers, matrix-related proteins, and matrix metallopeptidases (MMPs). These groups of genes are well known to be upregulated with mechanical loading and bone formation. Probes targeting the *Col1a1* gene were not detected on the exon array platform, so *Col1a1* expression was measured using qPCR. *Col1a1* expression in loaded ulnae followed a similar pattern as the genes in the matrix-formation (up) clusters. A few matrix genes were part of the matrix-formation (up) high-magnitude cluster, which had 23 total members and reached peak upregulation of approximately 4-fold at 12 days. *Lox* is important for collagen cross-linking, and *Cthrc1* and *Lepre1* are associated with collagen synthesis. In addition, this cluster included two proteoglycans, *Acan* and *Vcan*; a metallopeptidase inhibitor, *Timp1*; and two minor collagens, *Col5a1* and *Col11a2*.

The second most abundant gene group in the matrix-formation (up) clusters was comprised of solute carriers, which have not been previously associated with mechanical loading. Twenty solute carriers were upregulated by loading, and examples of the types of molecules transported include amino acids, glucose, and various ions. In addition, 15 solute carriers were downregulated during matrix formation. Solute carriers may be involved in supporting matrix synthesis by adjusting ion levels and bringing amino acids and other necessary ingredients into the osteoblasts to facilitate matrix protein production.

In addition to gene groups, we identified signaling pathways important in loading-induced bone formation. The Wnt/β-catenin signaling pathway is known to play an integral role in mechanotransduction and enhances the sensitivity of osteoblasts and osteocytes to loading.(45) The matrix-formation (up) clusters included several genes from the canonical Wnt pathway and also included two inhibitors, *Kremen1* and *Wif1.* Additionally, two Wnt inhibitors, *Sost* and *Sfrp4,* were downregulated during matrix formation. It was shown recently that *Sost* gene expression decreased after in vivo bone loading and increased with hind limb unloading.(15) Thus, decreased expression of *Sost* is associated with increased Wnt signaling and bone formation. SFRP4 has not been as well studied. The *Dkk1* gene encodes another Wnt antagonist that has been shown previously to decrease with loading,(15) but probes targeting the *Dkk1* gene were not detected on the exon array platform used in our study. We also observed increased *Osm* expression at early time points, and this has been shown to contribute to decreased *Sost* expression in bone.(34) LRP5 is a Wnt coreceptor, and Wnt signaling through LRP5 is required for mechanically induced bone formation.(45) In addition, it has been suggested that LRP5 may inhibit gut-derived serotonin synthesis, providing an endocrine regulatory mechanism to increase bone mass.(46)

Muscle genes were the largest group of downregulated genes during matrix formation. This group was comprised mainly of genes encoding muscle structural components and included members of the actin, myosin, and troponin gene families. It is important to note that all muscle was stripped from the ulnae during dissection, so RNA was isolated from bone and marrow only with no contamination from muscle. Therefore, these genes were expressed in cells within the bone. Others have identified muscle genes that were differentially expressed between osteoblasts and osteocytes.(47) Interestingly, 36 muscle-related genes were downregulated in osteoblasts with respect to osteocytes, including many of the same genes that were downregulated with loading in our study (*Acta1*, *Dmd*, *Myocd*, *Myl1*, *Myplf*, *Tnni2*, *Tnnt3*, and *Tpm2*).(47) We speculate that our finding that muscle-related genes are downregulated during bone formation is congruent with the finding that muscle-related genes are downregulated in osteoblasts compared with osteocytes. Although the involvement of muscle-related genes and proteins in bone biology is not well understood, it is clear that they are highly regulated in bone cells.

The TGF-β pathway is important in loading-induced bone formation,(48,49) and many genes in the TGF-β superfamily were upregulated during matrix formation. Two TGF-β superfamily inhibitor genes (*Chrdl1* and *Grem1*) were downregulated. Overall, it seems that signaling through the TGF-β pathway was enhanced mostly by increasing expression of TGF-β inducers and decreasing expression of TGF-β inhibitors of bone formation.

A limitation of our approach is that because we isolated RNA from the entire ulnar shaft, we cannot discern regional changes in bone formation in response to loading. It is well documented that mechanical loading elicits a regional bone-formation response in the ulna loading model.(4,14,50) Most of the bone forms in the ulna midshaft on the periosteal surface, and greater bone formation is observed on the medial surface than on the lateral surface.(14) In addition, since bone is deposited primarily on the medial and lateral periosteal surfaces in the ulnar midshaft, the geometric properties *I*_max_ and *I*_min_ change with loading and are location-dependent. *I*_min_ increased significantly in the ulnar midshaft, whereas *I*_max_ was moderately increased with loading distally.(4) In addition, we have completed a computational model that was programmed to form bone in regions that had greatest strain energy density.(50) This computational model almost perfectly mimicked the locations of new bone formation measured in the ulna, thus showing that there is a predictable pattern of bone formation in response to loading.

In conclusion, we determined the time sequence of gene expression in a bone subjected to mechanical loading, identified groups of genes that shared a time-dependent gene expression pattern, and determined which functions or pathways the genes had in common. We identified six clusters of genes that exhibited unique expression patterns in response to loading over a time course that captured key periods of the bone-formation process, including expression of matrix-related genes, the appearance of active osteoblasts, and bone desensitization. Many gene groups known to be important in loading-induced bone formation were identified within the clusters, including matrix, Wnt/β-catenin, and TGF-β. Several novel gene groups were identified as well, including chemokine, solute carrier, and muscle, whose functions with respect to bone formation are not known.
