# Supplementary material for: Gene Expression Patterns in Bone Following Mechanical Loading
Source: J Bone Miner Res. 2010 Jul 23;26(1):100–12. doi: 10.1002/jbmr.193 (PMC3179310; doi:10.1002/jbmr.193)
Supplement: Supplementary file 1 [file jbmr0026-0100-SD1.doc]

Supplementary Table 1. Complete list of differentially expressed genes in the early response cluster, the bone formation (up) clusters, and the bone formation (down) clusters. For the bone formation (up) clusters, H = high magnitude cluster, M = medium magnitude cluster, L = low magnitude cluster. For the bone formation (down) clusters, H = high magnitude cluster, L = low magnitude cluster.

| **Early Response Cluster** | | |  |
| --- | --- | --- | --- |
| **Gene Symbol** | **Gene Name** | |  |
| *A3galt2* | alpha 1,3-galactosyltransferase 2 | |  |
| *Adamts1* | ADAM metallopeptidase with thrombospondin type 1 motif | |  |
| *Adfp* | adipose differentiation related protein | |  |
| *Angptl4* | angiopoietin-like 4 | |  |
| *Ankh* | ankylosis, progressive homolog | |  |
| *Anxa2* | annexin A2 | |  |
| *Apold1* | apolipoprotein L domain containing 1 | |  |
| *Arf2* | ADP-ribosylation factor 2 | |  |
| *Arg1* | arginase, liver | |  |
| *C3ar1* | complement component 3a receptor 1 | |  |
| *Ccbp2* | chemokine binding protein 2 | |  |
| *Ccl2* | chemokine (C-C motif) ligand 2 | |  |
| *Ccl7* | chemokine (C-C motif) ligand 7 | |  |
| *Ccnd2* | cyclin d2 | |  |
| *Cd14* | CD14 molecule | |  |
| *Cfb* | complement factor B | |  |
| *Clic1* | chloride intracellular channel 1 | |  |
| *Crabp2* | cellular retinoic acid binding protein 2 | |  |
| *Crispld2* | cysteine-rich secretory protein LCCL domain containing 2 | |  |
| *Cst12* | cystatin 12 | |  |
| *Cxcl1* | chemokine (C-X-C motif) ligand 1 | |  |
| *Cxcl13* | chemokine (C-X-C motif) ligand 13 | |  |
| *Ecm1* | extracellular matrix protein 1 | |  |
| *Elovl6* | ELOVL family member 6, elongation of long chain fatty acids | |  |
| *Emp1* | epithelial membrane protein 1 | |  |
| *Enc1* | ectodermal-neural cortex 1 | |  |
| *Enpp3* | ectonucleotide pyrophosphatase/phosphodiesterase 3 | |  |
| *Entpd1* | ectonucleoside triphosphate diphosphohydrolase 1 | |  |
| *Fosl1* | fos-like antigen 1 | |  |
| *Fst* | follistatin | |  |
| *Gfpt2* | glutamine-fructose-6-phosphate transaminase 2 | |  |
| *Gpatch4* | G patch domain containing 4 | |  |
| *Gpr1* | G protein-coupled receptor 1 | |  |
| Supplementary Table 1 continued | | |  |
| **Early Response Cluster continued** | | |  |
| **Gene Symbol** | **Gene Name** | |  |
| *Hmga1* | high mobility group AT-hook 1 | |  |
| *Hmgcs1* | 3-hydroxy-3-methylglutaryl-Coenzyme A synthase 1 | |  |
| *Hsd11b1* | hydroxysteroid 11-beta dehydrogenase 1 | |  |
| *Hsd17b12* | hydroxysteroid (17-beta) dehydrogenase 12 | |  |
| *Hsd17b7* | hydroxysteroid (17-beta) dehydrogenase 7 | |  |
| *Hspa5* | heat shock protein 5 | |  |
| *Icam1* | intercellular adhesion molecule 1 | |  |
| *Ier2* | immediate early response 2 | |  |
| *Il1rl1* | interleukin 1 receptor-like 1 | |  |
| *Il1rl2* | interleukin 1 receptor-like 2 | |  |
| *Insig1* | insulin induced gene 1 | |  |
| *Junb* | jun B proto-oncogene | |  |
| *Kcne4* | potassium voltage-gated channel, Isk-related subfamily, gene 4 | |  |
| *Klrc3* | killer cell lectin-like receptor subfamily C, member 3 | |  |
| *Ldlr* | low density lipoprotein receptor | |  |
| *Lep* | leptin | |  |
| *Lilrb4* | leukocyte immunoglobulin-like receptor, subfamily B, member 4 | |  |
| *Lrat* | lecithin-retinol acyltransferase (phosphatidylcholine-retinol-O-acyltransferase) | |  |
| *Mall* | mal, T-cell differentiation protein-like | |  |
| *Mgl1* | macrophage galactose N-acetyl-galactosamine specific lectin 1 | |  |
| *Nos3* | nitric oxide synthase 3, endothelial cell | |  |
| *Olr1460* | olfactory receptor 1460 | |  |
| *Olr361* | olfactory receptor 361 | |  |
| *Olr436* | olfactory receptor 436 | |  |
| *Olr458* | olfactory receptor 458 | |  |
| *Olr811* | olfactory receptor 811 | |  |
| *Osm* | oncostatin M | |  |
| *Osmr* | oncostatin M receptor | |  |
| *Plp2* | proteolipid protein 2 (colonic epithelium-enriched) | |  |
| *Porf1* | preoptic regulatory factor 1 | |  |
| *Psat1* | phosphoserine aminotransferase 1 | |  |
| *PVR* | poliovirus receptor | |  |
| *RGD1308470* | similar to RIKEN cDNA 4933433P14 gene | |  |
| *S100a10* | S100 calcium binding protein A10 | |  |
| *S100a4* | S100 calcium binding protein A4 | |  |
| *Sc4mol* | sterol-C4-methyl oxidase-like | |  |
| *Sdc4* | syndecan 4 | |  |
| *Sectm1b* | secreted and transmembrane 1B | |  |
| *Sele* | selectin E | |  |
| *Selp* | selectin P | |  |
|  | | |  |
| Supplementary Table 1 continued | | |  |
| **Early Response Cluster continued** | | |  |
| **Gene Symbol** | **Gene Name** | |  |
| *Sema3a* | sema domain, immunoglobulin domain (Ig), short basic domain, secreted, (semaphorin) 3A | |  |
| *Serpina3n* | serine (or cysteine) peptidase inhibitor, clade A, member 3N | |  |
| *Serpine1* | serpin peptidase inhibitor, clade E, member 1 | |  |
| *Slc7a1* | solute carrier family 7 (cationic amino acid transporter, y+ system), member 1 | |  |
| *Slco2a1* | solute carrier organic anion transporter family, member 2a1 | |  |
| *Socs3* | suppressor of cytokine signaling 3 | |  |
| *Sqle* | squalene epoxidase | |  |
| *Stat3* | signal transducer and activator of transcription 3 | |  |
| *Tacr1* | tachykinin receptor 1 | |  |
| *Tfpi2* | tissue factor pathway inhibitor 2 | |  |
| *Tgm2* | transglutaminase 2, C polypeptide | |  |
| *Tnfrsf12a* | tumor necrosis factor receptor superfamily, member 12A | |  |
| *Trmt61a* | tRNA methyltransferase 61 homolog A | |  |
| *Ugdh* | UDP-glucose dehydrogenase | |  |
| *Zfp36* | zinc finger protein 36 | |  |
| **Matrix Formation (up) Clusters** | | |  |
| **Gene Symbol** | **Cluster** | **Gene Name** |  |
| *Aard* | L | alanine and arginine rich domain containing protein |  |
| *Abca1* | L | ATP-binding cassette, sub-family A (ABC1), member 1 |  |
| *Acan* | H | aggrecan | |
| *Acpl2* | H | acid phosphatase-like 2 | |
| *Adh7* | L | alcohol dehydrogenase 7 (class IV), mu or sigma polypeptide | |
| *Adra1a* | M | adrenergic, alpha-1A-, receptor | |
| *Agtr1b* | L | angiotensin II receptor, type 1b | |
| *Aifm3* | L | apoptosis-inducing factor, mitochondrion-associated 3 | |
| *Alpl* | M | alkaline phosphatase, liver/bone/kidney | |
| *Amigo3* | M | adhesion molecule with Ig like domain 3 | |
| *Angptl2* | M | angiopoietin-like 2 | |
| *Apba1* | L | amyloid beta (A4) precursor protein-binding, family A, member 1 | |
| *Apbb1* | L | amyloid beta (A4) precursor protein-binding, family B, member 1 | |
| *Apcs* | M | amyloid P component, serum | |
| *Apeg3* | L | antisense paternally expressed gene 3 | |
| *Apln* | L | apelin | |
| *Arf4* | M | ADP-ribosylation factor 4 | |
| *Arfip2* | L | ADP-ribosylation factor interacting protein 2 | |
| *Arl1* | L | ADP-ribosylation factor-like 1 | |
| *Armcx2* | L | armadillo repeat containing, X-linked 2 | |
| *Atp2b2* | L | ATPase, Ca++ transporting, plasma membrane 2 | |
| Supplementary Table 1 continued | | | |
| **Matrix Formation (up) Clusters continued** | | | |
| **Gene Symbol** | **Cluster** | **Gene Name** | |
| *Avpr2* | L | arginine vasopressin receptor 2 | |
| *Bambi* | M | BMP and activin membrane-bound inhibitor | |
| *Bcar1* | L | breast cancer anti-estrogen resistance 1 | |
| *Bcat1* | M | branched chain aminotransferase 1, cytosolic | |
| *Bet1* | L | blocked early in transport 1 homolog | |
| *Bex2* | L | brain expressed X-linked 2 | |
| *Bglap* | M | bone gamma-carboxyglutamate (gla) protein (osteocalcin) | |
| *Bgn* | M | biglycan | |
| *Bmp2* | L | Bone morphogenetic protein 2 | |
| *Bok* | L | BCL2-related ovarian killer | |
| *C1qtnf5* | M | C1q and tumor necrosis factor related protein 5 | |
| *Cacnb3* | M | calcium channel, voltage-dependent, beta 3 subunit | |
| *Cadps* | M | Ca++-dependent secretion activator | |
| *Calu* | M | calumenin | |
| *Capn6* | M | calpain 6 | |
| *Car8* | L | carbonic anhydrase 8 | |
| *Ccdc81* | L | coiled-coil domain containing 81 | |
| *Ccnd1* | M | cyclin D1 | |
| *Cd276* | L | Cd276 molecule | |
| *Cd63* | M | Cd63 molecule | |
| *Cdh15* | M | cadherin 15 | |
| *Cdh2* | M | cadherin 2 | |
| *Cdk5r1* | M | cyclin-dependent kinase 5, regulatory subunit 1 | |
| *Cdkn1a* | M | cyclin-dependent kinase inhibitor 1A | |
| *Cercam* | H | cerebral endothelial cell adhesion molecule | |
| *Cgref1* | H | cell growth regulator with EF hand domain 1 | |
| *Chgb* | L | chromogranin B | |
| *Chmp4c* | M | chromatin modifying protein 4C | |
| *Chn1* | M | chimerin (chimaerin) 1 | |
| *Chpf* | M | chondroitin polymerizing factor | |
| *Cit* | L | citron (rho-interacting, serine/threonine kinase 21) | |
| *Ckb* | L | creatine kinase, brain | |
| *Cldn5* | M | claudin 5 | |
| *Clec11a* | L | C-type lectin domain family 11, member a | |
| *Cntn1* | M | contactin 1 | |
| *Cntn3* | L | contactin 3 (plasmacytoma associated) | |
| *Col11a2* | H | collagen, type XI, alpha 2 | |
| *Col16a1* | M | collagen, type XVI, alpha 1 | |
| *Col1a2* | M | collagen, type I, alpha 2 | |
| *Col27a1* | L | collagen, type XXVII, alpha 1 | |
| *Col2a1* | M | collagen, type II, alpha 1 | |
| *Col3a1* | M | collagen, type III, alpha 1 | |
| Supplementary Table 1 continued | | | |
| **Matrix Formation (up) Clusters continued** | | | |
| **Gene Symbol** | **Cluster** | **Gene Name** | |
| *Col5a1* | H | collagen, type V, alpha 1 | |
| *Col5a3* | L | collagen, type V, alpha 3 | |
| *Copb1* | L | coatomer protein complex, subunit beta 1 | |
| *Cox4i2* | L | cytochrome c oxidase subunit IV isoform 2 | |
| *Cpz* | H | carboxypeptidase Z | |
| *Creb3l1* | H | cAMP responsive element binding protein 3-like 1 | |
| *Crtap* | M | cartilage associated protein | |
| *Cspg4* | L | chondroitin sulfate proteoglycan 4 | |
| *Cst8* | L | cystatin 8 (cystatin-related epididymal specific) | |
| *Cthrc1* | H | collagen triple helix repeat containing 1 | |
| *Cx3cr1* | L | chemokine (C-X3-C motif) receptor 1 | |
| *Cyb561d2* | L | cytochrome b-561 domain containing 2 | |
| *Cyb5d2* | L | cytochrome b5 domain containing 2 | |
| *Cybrd1* | M | cytochrome b reductase 1 | |
| *Cyp11b3* | L | cytochrome P450, subfamily 11B, polypeptide 3 | |
| *Cyp26b1* | L | cytochrome P450, family 26, subfamily b, polypeptide 1 | |
| *Dad1* | L | defender against cell death 1 | |
| *Dap* | L | death-associated protein | |
| *Dbn1* | L | drebrin 1 | |
| *Dbx1* | L | developing brain homeobox 1 | |
| *Ddost* | L | dolichyl-diphosphooligosaccharide-protein glycosyltransferase | |
| *Ddx25* | M | DEAD (Asp-Glu-Ala-Asp) box polypeptide 25 | |
| *Defb4* | L | defensin beta 4 | |
| *Dgkb* | L | diacylglycerol kinase, beta | |
| *Dlg4* | L | discs, large homolog 4 | |
| *Dnai2* | M | dynein, axonemal, intermediate chain 2 | |
| *Dnajb11* | L | DnaJ (Hsp40) homolog, subfamily B, member 11 | |
| *Dnm1* | L | dynamin 1 | |
| *Dpp6* | M | dipeptidylpeptidase 6 | |
| *Dpysl3* | M | dihydropyrimidinase-like 3 | |
| *Drd1a* | L | dopamine receptor D1A | |
| *Duox1* | M | dual oxidase 1 | |
| *Dync1i1* | L | dynein cytoplasmic 1 intermediate chain 1 | |
| *Efemp2* | L | EGF-containing fibulin-like extracellular matrix protein 2 | |
| *Efnb1* | L | ephrin B1 | |
| *Elavl2* | L | ELAV (embryonic lethal, abnormal vision, Drosophila)-like 2 (Hu antigen B) | |
| *Emp3* | L | epithelial membrane protein 3 | |
| *Enpp2* | L | ectonucleotide pyrophosphatase/phosphodiesterase 2 | |
| *Entpd3* | H | ectonucleoside triphosphate diphosphohydrolase 3 | |
| *Entpd5* | M | ectonucleoside triphosphate diphosphohydrolase 5 | |
| *Epb4.1l3* | L | erythrocyte protein band 4.1-like 3 | |
| Supplementary Table 1 continued | | | |
| **Matrix Formation (up) Clusters continued** | | | |
| **Gene Symbol** | **Cluster** | **Gene Name** | |
| *Erp29* | L | endoplasmic reticulum protein 29 | |
| *Fads1* | L | fatty acid desaturase 1 | |
| *Fads2* | M | fatty acid desaturase 2 | |
| *Fam101b* | L | family with sequence similarity 101, member B | |
| *Fam131b* | L | family with sequence similarity 131, member B | |
| *Fam89a* | L | family with sequence similarity 89, member A | |
| *Fam98a* | L | family with sequence similarity 98, member A | |
| *Fat3* | M | FAT tumor suppressor homolog 3 | |
| *Fbxl16* | L | F-box and leucine-rich repeat protein 16 | |
| *Fbxo16* | L | F-box protein 16 | |
| *Fev* | L | FEV (ETS oncogene family) | |
| *Fgf14* | L | fibroblast growth factor 14 | |
| *Fkbp10* | H | FK506 binding protein 10 | |
| *Fkbp11* | M | FK506 binding protein 11 | |
| *Fkbp14* | M | FK506 binding protein 14 | |
| *Flvcr2* | L | feline leukemia virus subgroup C cellular receptor family, member 2 | |
| *Fn1* | M | fibronectin 1 | |
| *Foxs1* | L | forkhead box S1 | |
| *Ftcd* | L | formiminotransferase cyclodeaminase | |
| *Fyn* | M | FYN oncogene related to SRC, FGR, YES | |
| *Gal* | M | galanin prepropeptide | |
| *Gale* | M | UDP-galactose-4-epimerase | |
| *Galk1* | L | galactokinase 1 | |
| *Galnt10* | L | UDP-N-acetyl-alpha-D-galactosamine:polypeptide N-acetylgalactosaminyltransferase 10 (GalNAc-T10) | |
| *Galnt3* | M | UDP-N-acetyl-alpha-D-galactosamine:polypeptide N-acetylgalactosaminyltransferase 3 (GalNAc-T3) | |
| *Galnt5* | M | UDP-N-acetyl-alpha-D-galactosamine:polypeptide N-acetylgalactosaminyltransferase 5 (GalNAc-T5) | |
| *Gcom1* | M | GRINL1A complex locus | |
| *Gdnf* | L | glial cell derived neurotrophic factor | |
| *Ggcx* | M | gamma-glutamyl carboxylase | |
| *Ggn* | L | gametogenetin | |
| *Gja1* | M | gap junction protein, alpha 1 | |
| *Gja6* | L | gap junction protein, alpha 6 | |
| *Gkn1* | L | gastrokine 1 | |
| *Glrb* | L | glycine receptor, beta | |
| *Glt8d1* | L | glycosyltransferase 8 domain containing 1 | |
| *Gmppa* | L | GDP-mannose pyrophosphorylase A | |
| *Gnai1* | M | guanine nucleotide binding protein (G protein), alpha inhibiting 1 | |
| Supplementary Table 1 continued | | | |
| **Matrix Formation (up) Clusters continued** | | | |
| **Gene Symbol** | **Cluster** | **Gene Name** | |
| *Gpr180* | L | G protein-coupled receptor 180 | |
| *Grin2d* | L | glutamate receptor, ionotropic, N-methyl D-aspartate 2D | |
| *Grin3a* | M | glutamate receptor, ionotropic, N-methyl-D-aspartate 3A | |
| *Gstm3* | L | glutathione S-transferase M3 | |
| *Gtf2ird1* | L | general transcription factor II I repeat domain-containing 1 | |
| *H1fnt* | L | H1 histone family, member N, testis-specific | |
| *Hapln1* | M | hyaluronan and proteoglycan link protein 1 | |
| *Hapln3* | M | hyaluronan and proteoglycan link protein 3 | |
| *Hdlbp* | M | high density lipoprotein binding protein (vigilin) | |
| *Hfe* | L | hemochromatosis | |
| *Hif1a* | L | hypoxia-inducible factor 1, alpha subunit | |
| *Hn1l* | L | hematological and neurological expressed 1-like | |
| *Hnf4a* | L | hepatocyte nuclear factor 4, alpha | |
| *Homer2* | M | homer homolog 2 | |
| *Hpcal1* | L | hippocalcin-like 1 | |
| *Hr* | L | hairless | |
| *Htr1d* | L | 5-hydroxytryptamine (serotonin) receptor 1D | |
| *Htr2a* | M | 5-hydroxytryptamine (serotonin) receptor 2A | |
| *Htra1* | L | HtrA serine peptidase 1 | |
| *Ibsp* | M | integrin binding sialoprotein | |
| *Id2* | L | inhibitor of DNA binding 2 | |
| *Id3* | M | inhibitor of DNA binding 3 | |
| *Igsf10* | M | immunoglobulin superfamily, member 10 | |
| *Il11* | L | interleukin 11 | |
| *Il12a* | M | interleukin 12a | |
| *Il4* | L | interleukin 4 | |
| *Inhba* | L | inhibin beta-A | |
| *Irs1* | M | insulin receptor substrate 1 | |
| *Jund* | L | Jun D proto-oncogene | |
| *Kcne3* | M | potassium voltage-gated channel, Isk-related subfamily, gene 3 | |
| *Kcng3* | L | potassium voltage-gated channel, subfamily G, member 3 | |
| *Kcnk6* | M | potassium inwardly-rectifying channel, subfamily K, member 6 | |
| *Kcnma1* | M | potassium large conductance calcium-activated channel, subfamily M, alpha member 1 | |
| *Kcnn2* | M | potassium intermediate/small conductance calcium-activated channel, subfamily N, member 2 | |
| *Kcns1* | L | potassium voltage-gated channel, delayed-rectifier, subfamily S, member 1 | |
| *Kdelr1* | M | KDEL (Lys-Asp-Glu-Leu) endoplasmic reticulum protein retention receptor 1 | |
| *Khdrbs2* | L | KH domain containing, RNA binding, signal transduction associated 2 | |
| Supplementary Table 1 continued | | | |
| **Matrix Formation (up) Clusters continued** | | | |
| **Gene Symbol** | **Cluster** | **Gene Name** | |
| *Kirrel* | L | kin of IRRE like | |
| *Klf4* | M | Kruppel-like factor 4 (gut) | |
| *Klks3* | L | kallikrein, submaxillary gland S3 | |
| *Kremen1* | M | kringle containing transmembrane protein 1 | |
| *Krt20* | L | keratin 20 | |
| *Krt27* | L | keratin 27 | |
| *Krt85* | L | keratin 85 | |
| *Lepre1* | H | leucine proline-enriched proteoglycan (leprecan) 1 | |
| *Lgals1* | M | lectin, galactoside-binding, soluble, 1 | |
| *Lgals2* | M | lectin, galactoside-binding, soluble 2 | |
| *Lgr4* | M | leucine-rich repeat-containing G protein-coupled receptor 4 | |
| *Lipc* | M | lipase, hepatic | |
| *Lman1* | M | lectin, mannose-binding, 1 | |
| *Lmna* | L | lamin A | |
| *LOC293989* | L | cytochrome P450-like | |
| *LOC299271* | M | serine proteinase inhibitor HongrES1 | |
| *LOC299282* | L | Serine protease inhibitor | |
| *LOC362526* | M | hypothetical protein LOC362526 | |
| *LOC362901* | L | Ac1254 | |
| *LOC364236* | L | similar to 40S ribosomal protein S19 | |
| *LOC499779* | L | similar to RIKEN cDNA 2900010J23 | |
| *LOC679840* | L | similar to germinal histone H4 gene | |
| *LOC691416* | L | hypothetical protein LOC691416 | |
| *Lox* | H | lysyl oxidase | |
| *Lpar3* | M | lysophosphatidic acid receptor 3 | |
| *Lphn1* | L | latrophilin 1 | |
| *Lppr2* | L | lipid phosphate phosphatase-related protein type 2 | |
| *Lrrc17* | H | leucine rich repeat containing 17 | |
| *Lrrc59* | M | leucine rich repeat containing 59 | |
| *Lrrn3* | L | leucine rich repeat neuronal 3 | |
| *Ltb4r2* | L | leukotriene B4 receptor 2 | |
| *Lxn* | L | latexin | |
| *Ly49i4* | L | Ly49 inhibitory receptor 4 | |
| *Maged1* | M | melanoma antigen, family D, 1 | |
| *Maged2* | M | melanoma antigen, family D, 2 | |
| *Magi2* | M | membrane associated guanylate kinase, WW and PDZ domain containing 2 | |
| *Marcksl1* | L | MARCKS-like 1 | |
| *Mbl2* | L | mannose binding lectin 2 (protein C) | |
| *Mdk* | L | midkine | |
| *Metrnl* | M | meteorin, glial cell differentiation regulator-like | |
| *MGC94199* | L | similar to RIKEN cDNA 2610301B20; EST AI428449 | |
| Supplementary Table 1 continued | | | |
| **Matrix Formation (up) Clusters continued** | | | |
| **Gene Symbol** | **Cluster** | **Gene Name** | |
| *Mia* | L | melanoma inhibitory activity | |
| *Mmp14* | M | matrix metallopeptidase 14 | |
| *Mmp16* | M | matrix metallopeptidase 16 | |
| *Mmp2* | M | matrix metallopeptidase 2 | |
| *Mmp23* | M | matrix metallopeptidase 23 | |
| *Mmp9* | M | matrix metallopeptidase 9 | |
| *Morf4l2* | M | mortality factor 4 like 2 | |
| *Msi1* | M | Musashi homolog 1 | |
| *Muc15* | M | mucin 15, cell surface associated | |
| *Myo1b* | M | myosin Ib | |
| *Myo5a* | M | myosin Va | |
| *Ndrg4* | L | N-myc downstream regulated gene 4 | |
| *Ndufa5* | L | NADH dehydrogenase (ubiquinone) 1 alpha subcomplex 5 | |
| *Neurod2* | L | neurogenic differentiation 2 | |
| *Ng23* | M | Ng23 protein | |
| *Ninj1* | M | ninjurin 1 | |
| *Nkx2-5* | L | NK2 transcription factor related, locus 5 | |
| *Nme1* | M | non-metastatic cells 1, protein (NM23A) expressed in | |
| *Nme2* | L | non-metastatic cells 2, protein (NM23B) expressed in | |
| *Nmur1* | M | neuromedin U receptor 1 | |
| *Npdc1* | L | neural proliferation, differentiation and control, 1 | |
| *Nr2f6* | L | nuclear receptor subfamily 2, group F, member 6 | |
| *Nr4a2* | L | nuclear receptor subfamily 4, group A, member 2 | |
| *Nrcam* | M | neuronal cell adhesion molecule | |
| *Nrep* | L | neuronal regeneration related protein | |
| *Nrm* | L | nurim (nuclear envelope membrane protein) | |
| *Nsg1* | M | neuron specific gene family member 1 | |
| *Nt5dc2* | M | 5'-nucleotidase domain containing 2 | |
| *Nupr1* | M | nuclear protein 1 | |
| *Oas1a* | M | 2'-5' oligoadenylate synthetase 1A | |
| *Odc1* | L | ornithine decarboxylase 1 | |
| *Olr1014* | L | olfactory receptor 1014 | |
| *Olr1055* | L | olfactory receptor 1055 | |
| *Olr110* | L | olfactory receptor 110 | |
| *Olr1105* | L | olfactory receptor 1105 | |
| *Olr1121* | L | olfactory receptor 1121 | |
| *Olr1129* | L | olfactory receptor 1129 | |
| *Olr1135* | L | olfactory receptor 1135 | |
| *Olr1204* | L | olfactory receptor 1204 | |
| *Olr1244* | L | olfactory receptor 1244 | |
| *Olr1253* | L | olfactory receptor 1253 | |
| *Olr1265* | L | olfactory receptor 1265 | |
| Supplementary Table 1 continued | | | |
| **Matrix Formation (up) Clusters continued** | | | |
| **Gene Symbol** | **Cluster** | **Gene Name** | |
| *Olr1273* | L | olfactory receptor 1273 | |
| *Olr1303* | L | olfactory receptor 1303 | |
| *Olr1325* | L | olfactory receptor 1325 | |
| *Olr1398* | L | olfactory receptor 1398 | |
| *Olr1409* | L | olfactory receptor 1409 | |
| *Olr1434* | L | olfactory receptor 1434 | |
| *Olr1563* | L | olfactory receptor 1563 | |
| *Olr1579* | L | olfactory receptor 1579 | |
| *Olr1600* | L | olfactory receptor 1600 | |
| *Olr1602* | L | olfactory receptor 1602 | |
| *Olr1646* | L | olfactory receptor 1646 | |
| *Olr1653* | L | olfactory receptor 1653 | |
| *Olr167* | L | olfactory receptor 167 | |
| *Olr168* | L | olfactory receptor 168 | |
| *Olr1751* | L | olfactory receptor 1751 | |
| *Olr180* | L | olfactory receptor 180 | |
| *Olr1l* | L | olfactory receptor 1-like | |
| *Olr259* | L | olfactory receptor 259 | |
| *Olr311* | L | olfactory receptor 311 | |
| *Olr346* | L | olfactory receptor 346 | |
| *Olr380* | L | olfactory receptor 380 | |
| *Olr434* | M | olfactory receptor 434 | |
| *Olr447* | L | olfactory receptor 447 | |
| *Olr448* | L | olfactory receptor 448 | |
| *Olr462* | L | olfactory receptor 462 | |
| *Olr53* | L | olfactory receptor 53 | |
| *Olr563* | L | olfactory receptor 563 | |
| *Olr6* | L | olfactory receptor 6 | |
| *Olr607* | L | olfactory receptor 607 | |
| *Olr614* | L | olfactory receptor 614 | |
| *Olr631* | L | olfactory receptor 631 | |
| *Olr661* | M | olfactory receptor 661 | |
| *Olr662* | L | olfactory receptor 662 | |
| *Olr696* | L | olfactory receptor 696 | |
| *Olr744* | L | olfactory receptor 744 | |
| *Olr749* | L | olfactory receptor 749 | |
| *Olr771* | L | olfactory receptor 771 | |
| *Olr839* | L | olfactory receptor 839 | |
| *Olr962* | L | olfactory receptor 962 | |
| *Omd* | M | osteomodulin | |
| *Onecut1* | L | one cut homeobox 1 | |
| *P22k15* | M | cystatin related protein 2 | |
| Supplementary Table 1 continued | | | |
| **Matrix Formation (up) Clusters continued** | | | |
| **Gene Symbol** | **Cluster** | **Gene Name** | |
| *P4ha1* | M | procollagen-proline, 2-oxoglutarate 4-dioxygenase (proline 4-hydroxylase), alpha polypeptide I | |
| *P4ha3* | M | procollagen-proline, 2-oxoglutarate 4-dioxygenase (proline 4-hydroxylase), alpha polypeptide III | |
| *P4hb* | M | prolyl 4-hydroxylase, beta polypeptide | |
| *Pacsin1* | M | protein kinase C and casein kinase substrate in neurons 1 | |
| *Panx3* | H | pannexin 3 | |
| *Pcdh8* | L | protocadherin 8 | |
| *Pcdhb2* | L | protocadherin beta 2 | |
| *Pcdhb5* | L | protocadherin beta 5 | |
| *Pcolce* | M | procollagen C-endopeptidase enhancer | |
| *Pcsk6* | M | proprotein convertase subtilisin/kexin type 6 | |
| *Pdc* | L | phosducin | |
| *Pde8a* | M | phosphodiesterase 8A | |
| *Pdgfa* | L | platelet-derived growth factor alpha polypeptide | |
| *Pdgfc* | M | platelet derived growth factor C | |
| *Pdgfrl* | M | platelet-derived growth factor receptor-like | |
| *Pdpn* | M | podoplanin | |
| *Pdzd2* | L | PDZ domain containing 2 | |
| *Pgf* | L | placental growth factor | |
| *Phex* | L | phosphate regulating gene with homologies to endopeptidases on the X chromosome (hypophosphatemia, vitamin D resistant rickets) | |
| *Phlda1* | L | pleckstrin homology-like domain, family A, member 1 | |
| *Plcd1* | M | phospholipase C, delta 1 | |
| *Plod2* | M | procollagen lysine, 2-oxoglutarate 5-dioxygenase 2 | |
| *Plod3* | M | procollagen-lysine, 2-oxoglutarate 5-dioxygenase 3 | |
| *Plvap* | L | plasmalemma vesicle associated protein | |
| *Ppib* | L | peptidylprolyl isomerase B | |
| *Ppic* | M | peptidylprolyl isomerase C | |
| *Ppm1e* | L | protein phosphatase 1E (PP2C domain containing) | |
| *Ppyr1* | L | pancreatic polypeptide receptor 1 | |
| *Prdx4* | M | peroxiredoxin 4 | |
| *Prelid1* | L | PRELI domain containing 1 | |
| *Prl8a5* | L | prolactin family 8, subfamily a, member 5 | |
| *Prl8a9* | L | prolactin family 8, subfamily a, member 9 | |
| *Prokr2* | L | prokineticin receptor 2 | |
| *Prrx1* | L | paired related homeobox 1 | |
| *Prss35* | H | protease, serine, 35 | |
| *Ptgfrn* | M | prostaglandin F2 receptor negative regulator | |
| *Ptgs2* | M | prostaglandin-endoperoxide synthase 2 | |
| *Pthr1* | M | parathyroid hormone receptor 1 | |
| Supplementary Table 1 continued | | | |
| **Matrix Formation (up) Clusters continued** | | | |
| **Gene Symbol** | **Cluster** | **Gene Name** | |
| *Ptn* | M | pleiotrophin | |
| *Ptprd* | L | protein tyrosine phosphatase, receptor type, D | |
| *Ptprk* | L | protein tyrosine phosphatase, receptor type, K, extracellular region | |
| *Ptprv* | M | protein tyrosine phosphatase, receptor type, V | |
| *Ptprz1* | L | protein tyrosine phosphatase, receptor-type, Z polypeptide 1 | |
| *Rab13* | M | RAB13, member RAS oncogene family | |
| *Rab34* | L | RAB34, member RAS oncogene family | |
| *Rab38* | L | RAB38, member RAS oncogene family | |
| *Rab4b* | L | RAB4B, member RAS oncogene family | |
| *Rap2ip* | L | Rap2 interacting protein | |
| *Rapgefl1* | L | Rap guanine nucleotide exchange factor (GEF)-like 1 | |
| *Rarres1* | M | retinoic acid receptor responder (tazarotene induced) 1 | |
| *rCG_22919* | L | hypothetical protein LOC100125364 | |
| *rCG_58138* | M | hypothetical protein LOC100125362 | |
| *Rcn3* | M | reticulocalbin 3, EF-hand calcium binding domain | |
| *Rell2* | L | RELT-like 2 | |
| *Rexo2* | L | REX2, RNA exonuclease 2 homolog | |
| *RGD1303130* | L | kidney predominant protein NCU-G1 | |
| *RGD1304952* | L | similar to RIKEN cDNA C530028O21 gene | |
| *RGD1305457* | M | similar to RIKEN cDNA 1700023M03 | |
| *RGD1307315* | L | LOC362793 | |
| *RGD1308059* | L | similar to DNA segment, Chr 4, Brigham & Womens Genetics 0951 expressed | |
| *Rgs19* | L | regulator of G-protein signaling 19 | |
| *Rin1* | L | Ras and Rab interactor 1 | |
| *Rit2* | L | Ras-like without CAAX 2 | |
| *Rmt1* | L | mammary cancer associated protein RMT-1 | |
| *Rnase11* | L | ribonuclease, RNase A family, 11 (non-active) | |
| *Rnase1l1* | L | ribonuclease, RNase A family, 1-like 1 (pancreatic) | |
| *Robo1* | M | roundabout homolog 1 | |
| *Rom1* | L | retinal outer segment membrane protein 1 | |
| *Rpl10* | L | ribosomal protein L10 | |
| *Rpl3* | L | ribosomal protein L3 | |
| *Rpl36al* | L | ribosomal protein L36a-like | |
| *Rpn1* | L | ribophorin I | |
| *Rpn2* | L | ribophorin II | |
| *RSA-14-44* | L | RSA-14-44 protein | |
| *Rsph1* | L | radial spoke head 1 homolog | |
| *RT1-CE3* | L | RT1 class I, CE3 | |
| *RT1-O* | L | RT1 class Ib, locus H2-Q-like, grc region | |
| *Rtn4rl2* | L | reticulon 4 receptor-like 2 | |
| Supplementary Table 1 continued | | | |
| **Matrix Formation (up) Clusters continued** | | | |
| **Gene Symbol** | **Cluster** | **Gene Name** | |
| *Sar1a* | M | SAR1 homolog A | |
| *Sbk1* | L | SH3-binding domain kinase 1 | |
| *Sc65* | M | synaptonemal complex protein SC65 | |
| *Scamp4* | L | secretory carrier membrane protein 4 | |
| *Scn1a* | L | sodium channel, voltage-gated, type I, alpha | |
| *Sct* | L | secretin | |
| *Sdc3* | M | syndecan 3 | |
| *Sec11c* | L | SEC11 homolog C | |
| *Sec16b* | M | SEC16 homolog B | |
| *Sec31a* | M | SEC31 homolog A | |
| *Sec61a1* | M | Sec61 alpha 1 subunit | |
| *Sels* | M | selenoprotein S | |
| *Sept5* | M | septin 5 | |
| *Serp1* | M | stress-associated endoplasmic reticulum protein 1 | |
| *Serpinb3* | L | serine protease inhibitor B3 | |
| *Serpinf1* | M | serine (or cysteine) peptidase inhibitor, clade F, member 1 | |
| *Serpinh1* | M | serine (or cysteine) peptidase inhibitor, clade H, member 1 | |
| *Serpini1* | L | serine (or cysteine) peptidase inhibitor, clade I, member 1 | |
| *Sfxn1* | L | sideroflexin 1 | |
| *Sgms2* | H | sphingomyelin synthase 2 | |
| *Sh3rf1* | L | SH3 domain containing ring finger 1 | |
| *Shank1* | L | SH3 and multiple ankyrin repeat domains 1 | |
| *Shroom2* | M | shroom family member 2 | |
| *Slc13a5* | H | solute carrier family 13 (sodium-dependent citrate transporter), member 5 | |
| *Slc1a4* | L | solute carrier family 1 (glutamate/neutral amino acid transporter), member 4 | |
| *Slc25a1* | L | solute carrier family 25 (mitochondrial carrier, citrate transporter), member 1 | |
| *Slc26a2* | L | solute carrier family 26 (sulfate transporter), member 2 | |
| *Slc2a13* | M | solute carrier family 2 (facilitated glucose transporter), member 13 | |
| *Slc30a4* | M | solute carrier family 30 (zinc transporter), member 4 | |
| *Slc31a1* | M | solute carrier family 31 (copper transporters), member 1 | |
| *Slc35b1* | L | solute carrier family 35, member B1 | |
| *Slc36a1* | M | solute carrier family 36 (proton/amino acid symporter), member 1 | |
| *Slc36a2* | H | solute carrier family 36 (proton/amino acid symporter), member 2 | |
| *Slc39a7* | M | solute carrier family 39 (zinc transporter), member 7 | |
| *Slc39a8* | M | solute carrier family 39 (metal ion transporter), member 8 | |
| *Slc5a5* | L | solute carrier family 5 (sodium iodide symporter), member 5 | |
| Supplementary Table 1 continued | | | |
| **Matrix Formation (up) Clusters continued** | | | |
| **Gene Symbol** | **Cluster** | **Gene Name** | |
| *Slc6a15* | M | solute carrier family 6 (neutral amino acid transporter), member 15 | |
| *Slc6a2* | M | solute carrier family 6 (neurotransmitter transporter, noradrenalin), member 2 | |
| *Slc7a8* | L | solute carrier family 7 (cationic amino acid transporter, y+ system), member 8 | |
| *Slc8a3* | M | solute carrier family 8 (sodium/calcium exchanger), member 3 | |
| *Slc9a2* | H | solute carrier family 9 (sodium/hydrogen exchanger), member 2 | |
| *Slc9a4* | M | solute carrier family 9 (sodium/hydrogen exchanger), member 4 | |
| *Slco3a1* | L | solute carrier organic anion transporter family, member 3a1 | |
| *Smad9* | M | SMAD family member 9 | |
| *Smcp* | L | sperm mitochondria-associated cysteine-rich protein | |
| *Smpd1* | M | sphingomyelin phosphodiesterase 1, acid lysosomal | |
| *Smpd3* | H | sphingomyelin phosphodiesterase 3, neutral | |
| *Snd1* | L | staphylococcal nuclease and tudor domain containing 1 | |
| *Snx7* | M | sorting nexin 7 | |
| *Socs2* | L | suppressor of cytokine signaling 2 | |
| *Sox11* | L | SRY (sex determining region Y)-box 11 | |
| *Sp7* | M | Sp7 transcription factor (osterix) | |
| *Sparc* | L | secreted protein, acidic, cysteine-rich (osteonectin) | |
| *Spink2* | L | serine peptidase inhibitor, Kazal type 2 (acrosin-trypsin inhibitor) | |
| *Spz1* | L | spermatogenic leucine zipper 1 | |
| *Sra1* | L | steroid receptor RNA activator 1 | |
| *Srm* | M | spermidine synthase | |
| *Srprb* | M | signal recognition particle receptor, B subunit | |
| *Srpx* | L | sushi-repeat-containing protein, X-linked | |
| *Ssr3* | L | signal sequence receptor, gamma | |
| *Ssr4* | M | signal sequence receptor, delta | |
| *Stc1* | L | stanniocalcin 1 | |
| *Steap3* | L | STEAP family member 3 | |
| *Stfa2l1* | L | stefin A2-like 1 | |
| *Syn1* | L | synapsin I | |
| *Syngap1* | L | synaptic Ras GTPase activating protein 1 homolog | |
| *Syt13* | L | synaptotagmin XIII | |
| *Syt3* | L | synaptotagmin III | |
| *Syt4* | M | synaptotagmin IV | |
| *Taar6* | L | trace amine-associated receptor 6 | |
| *Tacr3* | L | tachykinin receptor 3 | |
| *Tanc1* | L | tetratricopeptide repeat, ankyrin repeat and coiled-coil containing 1 | |
| *Tas2r118* | L | taste receptor, type 2, member 118 | |
| Supplementary Table 1 continued | | | |
| **Matrix Formation (up) Clusters continued** | | | |
| **Gene Symbol** | **Cluster** | **Gene Name** | |
| *Tbc1d20* | L | TBC1 domain family, member 20 | |
| *Tdg* | L | thymine-DNA glycosylase | |
| *Tessp6* | L | testis-specific serine protease-6 | |
| *Tex264* | M | testis expressed 264 | |
| *Tff3* | M | trefoil factor 3, intestinal | |
| *Tfg* | M | Trk-fused gene | |
| *Tgfb1* | L | transforming growth factor, beta 1 | |
| *Tgfb3* | L | transforming growth factor, beta 3 | |
| *Thtpa* | L | thiamine triphosphatase | |
| *Thy1* | L | Thy-1 cell surface antigen | |
| *Timp1* | H | TIMP metallopeptidase inhibitor 1 | |
| *Tmed3* | M | transmembrane emp24 protein transport domain containing 3 | |
| *Tmed9* | L | transmembrane emp24 protein transport domain containing 9 | |
| *Tmem120a* | M | transmembrane protein 120A | |
| *Tmem132a* | L | transmembrane protein 132A | |
| *Tmem214* | L | transmembrane protein 214 | |
| *Tmem35* | L | transmembrane protein 35 | |
| *Tmem37* | L | transmembrane protein 37 | |
| *Tmem39a* | M | transmembrane protein 39a | |
| *Tmem97* | M | transmembrane protein 97 | |
| *Tmem98* | L | transmembrane protein 98 | |
| *Tnp2* | L | transition protein 2 | |
| *Tor2a* | L | torsin family 2, member A | |
| *Tpm4* | L | tropomyosin 4 | |
| *Tpo1* | M | developmentally regulated protein TPO1 | |
| *Tpst2* | L | tyrosylprotein sulfotransferase 2 | |
| *Tram1* | M | translocation associated membrane protein 1 | |
| *Trpv4* | M | transient receptor potential cation channel, subfamily V, member 4 | |
| *Tsku* | M | tsukushin | |
| *Ttc36* | L | tetratricopeptide repeat domain 36 | |
| *Tuba3a* | L | tubulin, alpha 3A | |
| *Tusc3* | M | tumor suppressor candidate 3 | |
| *Twf1* | M | twinfilin, actin-binding protein, homolog 1 | |
| *Uba5* | L | ubiquitin-like modifier activating enzyme 5 | |
| *Ugt2b5* | L | UDP glucuronosyltransferase 2 family, polypeptide B5 | |
| *Unc5b* | M | unc-5 homolog B | |
| *Unc5c* | M | unc-5 homolog C | |
| *Uncx* | L | UNC homeobox | |
| *Uso1* | L | USO1 homolog, vesicle docking protein | |
| *Uxs1* | L | UDP-glucuronate decarboxylase 1 | |
| *V1rc29* | L | vomeronasal 1 receptor, C29 | |
| Supplementary Table 1 continued | | | |
| **Matrix Formation (up) Clusters continued** | | | |
| **Gene Symbol** | **Cluster** | **Gene Name** | |
| *V1rd25* | L | vomeronasal 1 receptor, D25 | |
| *V1rf1* | L | vomeronasal 1 receptor, F1 | |
| *Vcan* | H | versican | |
| *Vdr* | L | vitamin D (1,25-dihydroxyvitamin D3) receptor | |
| *Vkorc1* | M | vitamin K epoxide reductase complex, subunit 1 | |
| *Wfdc1* | L | WAP four-disulfide core domain 1 | |
| *Wfdc12* | L | WAP four-disulfide core domain 12 | |
| *Wfdc9* | L | WAP four-disulfide core domain 9 | |
| *Wif1* | M | Wnt inhibitory factor 1 | |
| *Wisp1* | M | WNT1 inducible signaling pathway protein 1 | |
| *Xkr4* | L | XK, Kell blood group complex subunit-related family, member 4 | |
| *Yif1* | L | Yip1 interacting factor homolog | |
| *Yif1b* | L | Yip1 interacting factor homolog B | |
| *Ykt6* | M | YKT6 v-SNARE homolog | |
| *Zcchc12* | M | zinc finger, CCHC domain containing 12 | |
| *Zfp260* | L | zinc finger protein 260 | |
| *Zfp354c* | M | zinc finger protein 354C | |
| *Zfp367* | L | zinc finger protein 367 | |
| **Matrix Formation (down) Clusters** | | | |
| **Gene Symbol** | **Cluster** | **Gene Name** | |
| *Abcb1* | H | ATP-binding cassette, sub-family B (MDR/TAP), member 1 | |
| *Abcb10* | L | ATP-binding cassette, sub-family B (MDR/TAP), member 10 | |
| *Abcb4* | L | ATP-binding cassette, sub-family B (MDR/TAP), member 4 | |
| *Acaa2* | L | acetyl-Coenzyme A acyltransferase 2 | |
| *Acacb* | H | acetyl-Coenzyme A carboxylase beta | |
| *Acadm* | L | acyl-Coenzyme A dehydrogenase, C-4 to C-12 straight chain | |
| *Acsl1* | L | acyl-CoA synthetase long-chain family member 1 | |
| *Acsl6* | H | acyl-CoA synthetase long-chain family member 6 | |
| *Acta1* | H | actin, alpha 1 | |
| *Actn3* | H | actinin alpha 3 | |
| *Add3* | L | adducin 3 (gamma) | |
| *Adprhl1* | H | ADP-ribosylhydrolase like 1 | |
| *Adra1b* | L | adrenergic, alpha-1B-, receptor | |
| *Adrb2* | H | adrenergic, beta-2-, receptor, surface | |
| *Akap12* | L | A kinase (PRKA) anchor protein 12 | |
| *Akap6* | H | A kinase (PRKA) anchor protein 6 | |
| *Akap7* | L | A kinase (PRKA) anchor protein 7 | |
| *Akr1c14* | L | aldo-keto reductase family 1, member C14 | |
| *Alcam* | L | activated leukocyte cell adhesion molecule | |
| *Aldh1a2* | H | aldehyde dehydrogenase 1 family, member A2 | |
| *Aldh3a1* | L | aldehyde dehydrogenase 3 family, member A1 | |
| *Aldoa* | L | aldolase A, fructose-bisphosphate | |
| Supplementary Table 1 continued | | | |
| **Matrix Formation (down) Clusters continued** | | | |
| **Gene Symbol** | **Cluster** | **Gene Name** | |
| *Alox5* | L | arachidonate 5-lipoxygenase | |
| *Amn1* | L | antagonist of mitotic exit network 1 homolog (S. cerevisiae) | |
| *Ampd1* | H | adenosine monophosphate deaminase 1 (isoform M) | |
| *Ampd3* | L | adenosine monophosphate deaminase 3 | |
| *Angpt1* | L | angiopoietin 1 | |
| *Ank3* | L | ankyrin 3, epithelial | |
| *Anp32e* | L | acidic (leucine-rich) nuclear phosphoprotein 32 family, member E | |
| *Anxa1* | L | annexin A1 | |
| *Anxa3* | L | annexin A3 | |
| *Apobec1* | L | apolipoprotein B mRNA editing enzyme, catalytic polypeptide 1 | |
| *Aqp4* | H | aquaporin 4 | |
| *Arhgap15* | L | Rho GTPase activating protein 15 | |
| *Arhgdib* | L | Rho, GDP dissociation inhibitor (GDI) beta | |
| *Arl6ip1* | L | ADP-ribosylation factor-like 6 interacting protein 1 | |
| *Arrb2* | L | arrestin, beta 2 | |
| *Asb2* | H | ankyrin repeat and SOCS box-containing 2 | |
| *Asph* | H | aspartate-beta-hydroxylase | |
| *Atp1a2* | H | ATPase, Na+/K+ transporting, alpha 2 polypeptide | |
| *Atp1b1* | H | ATPase, Na+/K+ transporting, beta 1 polypeptide | |
| *Atp1b4* | L | ATPase, (Na+)/K+ transporting, beta 4 polypeptide | |
| *Atp5a1* | L | ATP synthase, H+ transporting, mitochondrial F1 complex, alpha subunit 1, cardiac muscle | |
| *Atp5b* | L | ATP synthase, H+ transporting, mitochondrial F1 complex, beta polypeptide | |
| *Atp5f1* | L | ATP synthase, H+ transporting, mitochondrial F0 complex, subunit B1 | |
| *Atp5s* | L | ATP synthase, H+ transporting, mitochondrial F0 complex, subunit s (factor B) | |
| *Atp7b* | L | ATPase, Cu++ transporting, beta polypeptide | |
| *Aurka* | L | aurora kinase A | |
| *B4galnt1* | L | beta-1,4-N-acetyl-galactosaminyl transferase 1 | |
| *B4galt6* | L | UDP-Gal:betaGlcNAc beta 1,4-galactosyltransferase, polypeptide 6 | |
| *Bcap29* | L | B-cell receptor-associated protein 29 | |
| *Bcl11a* | L | B-cell CLL/lymphoma 11A (zinc finger protein) | |
| *Bin1* | H | bridging integrator 1 | |
| *Bmpr1b* | H | bone morphogenetic protein receptor, type IB | |
| *Bpgm* | L | 2,3-bisphosphoglycerate mutase | |
| *Brca1* | L | breast cancer 1 | |
| *Btk* | L | Bruton agammaglobulinemia tyrosine kinase | |
| *Btla* | L | B and T lymphocyte associated | |
| Supplementary Table 1 continued | | | |
| **Matrix Formation (down) Clusters continued** | | | |
| **Gene Symbol** | **Cluster** | **Gene Name** | |
| *C3* | L | complement component 3 | |
| *C6* | L | complement component 6 | |
| *Cab39l* | L | calcium binding protein 39-like | |
| *Cabc1* | H | chaperone, ABC1 activity of bc1 complex homolog (S. pombe) | |
| *Cacnb1* | H | calcium channel, voltage-dependent, beta 1 subunit | |
| *Cacng1* | H | calcium channel, voltage-dependent, gamma subunit 1 | |
| *Cacng7* | H | calcium channel, voltage-dependent, gamma subunit 7 | |
| *Calb2* | H | calbindin 2 | |
| *Camk2b* | H | calcium/calmodulin-dependent protein kinase (CaM kinase) II beta | |
| *Capn3* | H | calpain 3 | |
| *Car2* | L | carbonic anhydrase II | |
| *Casp1* | L | caspase 1 | |
| *Casq2* | H | calsequestrin 2 | |
| *Cbx7* | L | chromobox homolog 7 | |
| *Ccdc82* | L | coiled-coil domain containing 82 | |
| *Ccl11* | L | chemokine (C-C motif) ligand 11 | |
| *Ccna2* | L | cyclin A2 | |
| *Ccnb1* | L | cyclin B1 | |
| *Ccr1* | L | chemokine (C-C motif) receptor 1 | |
| *Ccr2* | L | chemokine (C-C motif) receptor 2 | |
| *Ccr3* | L | chemokine (C-C motif) receptor 3 | |
| *Cd37* | L | CD37 molecule | |
| *Cd53* | L | Cd53 molecule | |
| *Cdadc1* | L | cytidine and dCMP deaminase domain containing 1 | |
| *Cdc25b* | L | cell division cycle 25 homolog B | |
| *Cdh1* | L | cadherin 1 | |
| *Cdh19* | H | cadherin 19, type 2 | |
| *Ceacam1* | L | carcinoembryonic antigen-related cell adhesion molecule 1 (biliary glycoprotein) | |
| *Cenpc1* | L | centromere protein C 1 | |
| *Cenpi* | L | centromere protein I | |
| *Cep70* | L | centrosomal protein 70kDa | |
| *Chi3l1* | L | chitinase 3-like 1 | |
| *Chrdl1* | L | chordin-like 1 | |
| *Chrna1* | H | cholinergic receptor, nicotinic, alpha 1 | |
| *Chrnb1* | H | cholinergic receptor, nicotinic, beta 1 | |
| *Chrne* | H | cholinergic receptor, nicotinic, epsilon | |
| *Cited4* | L | Cbp/p300-interacting transactivator, with Glu/Asp-rich carboxy-terminal domain, 4 | |
| *Ckm* | H | creatine kinase, muscle | |
| *Clca2* | L | chloride channel calcium activated 2 | |
| Supplementary Table 1 continued | | | |
| **Matrix Formation (down) Clusters continued** | | | |
| **Gene Symbol** | **Cluster** | **Gene Name** | |
| *Cldn1* | H | claudin 1 | |
| *Clec4d* | L | C-type lectin domain family 4, member d | |
| *Clic5* | L | chloride intracellular channel 5 | |
| *Clip4* | H | CAP-GLY domain containing linker protein family, member 4 | |
| *Cmbl* | H | carboxymethylenebutenolidase homolog (Pseudomonas) | |
| *Cntfr* | L | ciliary neurotrophic factor receptor | |
| *Coq3* | L | coenzyme Q3 homolog, methyltransferase (S. cerevisiae) | |
| *Coro1a* | L | coronin, actin binding protein 1A | |
| *Cox6a2* | H | cytochrome c oxidase, subunit VIa, polypeptide 2 | |
| *Cpt1b* | H | carnitine palmitoyltransferase 1b, muscle | |
| *Csf1* | L | colony stimulating factor 1 (macrophage) | |
| *Csrp3* | H | cysteine and glycine-rich protein 3 | |
| *Ctse* | L | cathepsin E | |
| *Cugbp2* | L | CUG triplet repeat, RNA binding protein 2 | |
| *Cxcl12* | L | chemokine (C-X-C motif) ligand 12 | |
| *Cxcl14* | H | chemokine (C-X-C motif) ligand 14 | |
| *Cxcr5* | L | chemokine (C-X-C motif) receptor 5 | |
| *Cybb* | L | cytochrome b-245, beta polypeptide | |
| *Cyp2e1* | L | cytochrome P450, family 2, subfamily e, polypeptide 1 | |
| *Cyp2j4* | H | cytochrome P450, family 2, subfamily J, polypeptide 4 | |
| *Cytip* | L | cytohesin 1 interacting protein | |
| *Dck* | L | deoxycytidine kinase | |
| *Des* | H | desmin | |
| *Dld* | L | dihydrolipoamide dehydrogenase | |
| *Dmd* | H | dystrophin, muscular dystrophy | |
| *Dnase1l3* | L | deoxyribonuclease 1-like 3 | |
| *Dpp4* | L | dipeptidylpeptidase 4 | |
| *Dpyd* | L | dihydropyrimidine dehydrogenase | |
| *Dusp13* | L | dual specificity phosphatase 13 | |
| *Dusp26* | L | dual specificity phosphatase 26 (putative) | |
| *Dusp6* | L | dual specificity phosphatase 6 | |
| *Eef1a2* | H | eukaryotic translation elongation factor 1 alpha 2 | |
| *Eepd1* | L | endonuclease/exonuclease/phosphatase family domain containing 1 | |
| *Efemp1* | H | EGF-containing fibulin-like extracellular matrix protein 1 | |
| *Egf* | L | epidermal growth factor | |
| *Egln3* | L | EGL nine homolog 3 (C. elegans) | |
| *Egr1* | L | early growth response 1 | |
| *Egr3* | H | early growth response 3 | |
| *Eif3e* | L | eukaryotic translation initiation factor 3, subunit E | |
| *Emb* | L | embigin | |
|  | | | |
| Supplementary Table 1 continued | | | |
| **Matrix Formation (down) Clusters continued** | | | |
| **Gene Symbol** | **Cluster** | **Gene Name** | |
| *Emr4p* | L | EGF-like module containing, mucin-like, hormone receptor-like 4 | |
| *Eno3* | H | enolase 3, beta, muscle | |
| *Ensa* | L | endosulfine alpha | |
| *Epha7* | L | Eph receptor A7 | |
| *Ephx1* | L | epoxide hydrolase 1, microsomal | |
| *Errfi1* | L | ERBB receptor feedback inhibitor 1 | |
| *Esm1* | L | endothelial cell-specific molecule 1 | |
| *F2rl2* | L | coagulation factor II (thrombin) receptor-like 2 | |
| *Fabp3* | H | fatty acid binding protein 3, muscle and heart | |
| *Fam107b* | L | family with sequence similarity 107, member B | |
| *Fancd2* | L | Fanconi anemia, complementation group D2 | |
| *Far1* | L | fatty acyl CoA reductase 1 | |
| *Fastkd2* | L | FAST kinase domains 2 | |
| *Fbp2* | H | fructose-1,6-bisphosphatase 2 | |
| *Fbxo32* | H | F-box protein 32 | |
| *Fcgr2b* | L | Fc fragment of IgG, low affinity IIb, receptor (CD32) | |
| *Fcgr3* | L | Fc receptor, IgG, low affinity III | |
| *Fcn1* | L | ficolin (collagen/fibrinogen domain containing) 1 | |
| *Fgf1* | L | fibroblast growth factor 1 | |
| *Fgf23* | H | fibroblast growth factor 23 | |
| *Fgf7* | H | fibroblast growth factor 7 | |
| *Fggy* | L | FGGY carbohydrate kinase domain containing | |
| *Fgl2* | H | fibrinogen-like 2 | |
| *Fgr* | L | Gardner-Rasheed feline sarcoma viral (v-fgr) oncogene homolog | |
| *Fhl1* | H | four and a half LIM domains 1 | |
| *Fignl1* | L | fidgetin-like 1 | |
| *Filip1* | H | filamin A interacting protein 1 | |
| *Fkbp5* | L | FK506 binding protein 5 | |
| *Fmo1* | L | flavin containing monooxygenase 1 | |
| *Fmo2* | H | flavin containing monooxygenase 2 | |
| *Fnbp1* | L | formin binding protein 1 | |
| *Fut2* | L | fucosyltransferase 2 (secretor status included) | |
| *Fxyd1* | L | FXYD domain-containing ion transport regulator 1 | |
| *Gadd45a* | L | growth arrest and DNA-damage-inducible, alpha | |
| *Galc* | L | galactosylceramidase | |
| *Gas6* | L | growth arrest specific 6 | |
| *Gbas* | L | glioblastoma amplified sequence | |
| *Gclm* | L | glutamate cysteine ligase, modifier subunit | |
| *Gfra1* | L | GDNF family receptor alpha 1 | |
| *Ghitm* | L | growth hormone inducible transmembrane protein | |
| Supplementary Table 1 continued | | | |
| **Matrix Formation (down) Clusters continued** | | | |
| **Gene Symbol** | **Cluster** | **Gene Name** | |
| *Ghr* | L | growth hormone receptor | |
| *Gimap7* | L | GTPase, IMAP family member 7 | |
| *Glipr1* | L | GLI pathogenesis-related 1 | |
| *Gmpr* | H | guanosine monophosphate reductase | |
| *Got1* | H | glutamic-oxaloacetic transaminase 1, soluble (aspartate aminotransferase 1) | |
| *Got2* | L | glutamic-oxaloacetic transaminase 2, mitochondrial (aspartate aminotransferase 2) | |
| *Gpc3* | L | glypican 3 | |
| *Gpi* | L | glucose phosphate isomerase | |
| *Gpr116* | L | G protein-coupled receptor 116 | |
| *Gpt2* | H | glutamic pyruvate transaminase (alanine aminotransferase) 2 | |
| *Gpx1* | L | glutathione peroxidase 1 | |
| *Grem1* | H | gremlin 1, cysteine knot superfamily | |
| *Gsr* | L | glutathione reductase | |
| *Gsta3* | L | glutathione S-transferase A3 | |
| *Gstm1* | L | glutathione S-transferase M1 | |
| *Gstm2* | L | glutathione S-transferase M2 | |
| *Gstm7* | H | glutathione S-transferase, mu 7 | |
| *Gyg1* | H | glycogenin 1 | |
| *Hat1* | L | histone acetyltransferase 1 | |
| *Hba-a2* | L | hemoglobin alpha, adult chain 2 | |
| *Hbb* | L | hemoglobin, beta | |
| *Hcn4* | L | hyperpolarization activated cyclic nucleotide-gated potassium channel 4 | |
| *Hdc* | L | histidine decarboxylase | |
| *Hemgn* | L | hemogen | |
| *Hfe2* | H | hemochromatosis type 2 (juvenile) (human homolog) | |
| *Hgf* | L | hepatocyte growth factor | |
| *Hipk3* | L | homeodomain interacting protein kinase 3 | |
| *Hmbs* | L | hydroxymethylbilane synthase | |
| *Hprt1* | L | hypoxanthine phosphoribosyltransferase 1 | |
| *Hrc* | H | histidine rich calcium binding protein | |
| *Hspa9* | L | heat shock protein 9 | |
| *Hspb6* | H | heat shock protein, alpha-crystallin-related, B6 | |
| *Hspb8* | H | heat shock protein 8 | |
| *Htr1b* | L | 5-hydroxytryptamine (serotonin) receptor 1B | |
| *Idh3a* | H | isocitrate dehydrogenase 3 (NAD+) alpha | |
| *Igfbp6* | L | insulin-like growth factor binding protein 6 | |
| *Il8rb* | L | interleukin 8 receptor, beta | |
| *Ipo13* | L | importin 13 | |
| *Irf8* | L | interferon regulatory factor 8 | |
| Supplementary Table 1 continued | | | |
| **Matrix Formation (down) Clusters continued** | | | |
| **Gene Symbol** | **Cluster** | **Gene Name** | |
| *Itgad* | L | integrin, alpha D | |
| *Itgam* | L | integrin alpha M | |
| *Itgb4* | H | integrin beta 4 | |
| *Kars* | L | lysyl-tRNA synthetase | |
| *Kcnj11* | H | potassium inwardly rectifying channel, subfamily J, member 11 | |
| *Kcnj12* | H | potassium inwardly-rectifying channel, subfamily J, member 12 | |
| *Kcnj3* | L | potassium inwardly-rectifying channel, subfamily J, member 3 | |
| *Kcnn4* | L | potassium intermediate/small conductance calcium-activated channel, subfamily N, member 4 | |
| *Kif15* | L | kinesin family member 15 | |
| *Kit* | L | v-kit Hardy-Zuckerman 4 feline sarcoma viral oncogene homolog | |
| *Kitlg* | L | KIT ligand | |
| *Klf15* | L | Kruppel-like factor 15 | |
| *Klra2* | L | killer cell lectin-like receptor, subfamily A, member 2 | |
| *Kng1* | L | kininogen 1 | |
| *Kpna1* | L | karyopherin (importin) alpha 1 | |
| *Krt19* | H | keratin 19 | |
| *Kynu* | L | kynureninase (L-kynurenine hydrolase) | |
| *Lbr* | L | lamin B receptor | |
| *Lcp1* | L | lymphocyte cytosolic protein 1 | |
| *Ldb3* | H | LIM domain binding 3 | |
| *Ldha* | L | lactate dehydrogenase A | |
| *Lepr* | L | leptin receptor | |
| *Lgi4* | L | leucine-rich repeat LGI family, member 4 | |
| *Lias* | L | lipoic acid synthetase | |
| *Lmnb1* | L | lamin B1 | |
| *LOC24906* | H | RoBo-1 | |
| *LOC287167* | L | globin, alpha | |
| *LOC298139* | H | similar to RIKEN cDNA 2310003M01 | |
| *LOC498265* | L | similar to hypothetical protein FLJ10706 | |
| *LOC501110* | L | similar to Glutathione S-transferase A1 (GTH1) (HA subunit 1) (GST-epsilon) (GSTA1-1) (GST class-alpha) | |
| *Lphn3* | L | latrophilin 3 | |
| *Lpin1* | L | lipin 1 | |
| *Lrrc2* | H | leucine rich repeat containing 2 | |
| *Ly6c* | L | Ly6-C antigen | |
| *Lyz2* | L | lysozyme 2 | |
| *Macrod1* | H | MACRO domain containing 1 | |
| *Mal* | L | mal, T-cell differentiation protein | |
| *Maob* | L | monoamine oxidase B | |
| *Mapk12* | H | mitogen-activated protein kinase 12 | |
| Supplementary Table 1 continued | | | |
| **Matrix Formation (down) Clusters continued** | | | |
| **Gene Symbol** | **Cluster** | **Gene Name** | |
| *Mapt* | L | microtubule-associated protein tau | |
| *Mb* | H | myoglobin | |
| *Mbp* | L | myelin basic protein | |
| *Mcm7* | L | minichromosome maintenance deficient 7 (S. cerevisiae) | |
| *Mcpt1* | L | mast cell protease 1 | |
| *Mdh1* | L | malate dehydrogenase 1, NAD (soluble) | |
| *MGC105649* | L | hypothetical LOC302884 | |
| *MGC109340* | L | similar to Microsomal signal peptidase 23 kDa subunit (SPase 22 kDa subunit) (SPC22/23) | |
| *MGC72973* | L | beta-glo | |
| *MGC95152* | L | similar to B230212L03Rik protein | |
| *Mkrn1* | L | makorin ring finger protein 1 | |
| *Mmp8* | L | matrix metallopeptidase 8 | |
| *Mrps18b* | L | mitochondrial ribosomal protein S18B | |
| *Ms4a2* | L | membrane-spanning 4-domains, subfamily A, member 2 (Fc fragment of IgE, high affinity I, receptor for; beta polypeptide) | |
| *Ms4a6b* | L | membrane-spanning 4-domains, subfamily A, member 6B | |
| *Mustn1* | L | musculoskeletal, embryonic nuclear protein 1 | |
| *Mx1* | L | myxovirus (influenza virus) resistance 1 | |
| *Myl1* | H | myosin, light polypeptide 1 | |
| *Mylk2* | H | myosin light chain kinase 2 | |
| *Mylpf* | H | myosin light chain, phosphorylatable, fast | |
| *Myoc* | H | myocilin | |
| *Myocd* | H | myocardin | |
| *Ndrg1* | L | N-myc downstream regulated gene 1 | |
| *Ndrg2* | H | NDRG family member 2 | |
| *Ndufs1* | H | NADH dehydrogenase (ubiquinone) Fe-S protein 1 | |
| *Ndufs4* | L | NADH dehydrogenase (ubiquinone) Fe-S protein 4 | |
| *Nexn* | H | nexilin (F actin binding protein) | |
| *Nmnat3* | L | nicotinamide nucleotide adenylyltransferase 3 | |
| *Nqo1* | L | NAD(P)H dehydrogenase, quinone 1 | |
| *Nr3c2* | L | nuclear receptor subfamily 3, group C, member 2 | |
| *Ntf3* | L | neurotrophin 3 | |
| *Ntn1* | H | netrin 1 | |
| *Nuf2* | L | NUF2, NDC80 kinetochore complex component, homolog (S. cerevisiae) | |
| *Nup210* | L | nucleoporin 210 | |
| *Nup35* | L | nucleoporin 35 | |
| *Oas1k* | L | 2 ' -5 ' oligoadenylate synthetase 1K | |
| *Obfc2a* | L | oligonucleotide/oligosaccharide-binding fold containing 2A | |
| *Odz2* | L | odz, odd Oz/ten-m homolog 2 (Drosophila) | |
| *Olr1372* | L | olfactory receptor 1372 | |
| Supplementary Table 1 continued | | | |
| **Matrix Formation (down) Clusters continued** | | | |
| **Gene Symbol** | **Cluster** | **Gene Name** | |
| *Olr325* | L | olfactory receptor 325 | |
| *Oxr1* | L | oxidation resistance 1 | |
| *P2ry1* | L | purinergic receptor P2Y, G-protein coupled 1 | |
| *Pacsin3* | L | protein kinase C and casein kinase substrate in neurons 3 | |
| *Pc* | L | pyruvate carboxylase | |
| *Pcmt1* | L | protein-L-isoaspartate (D-aspartate) O-methyltransferase 1 | |
| *Pcsk1* | L | proprotein convertase subtilisin/kexin type 1 | |
| *Pde4b* | L | phosphodiesterase 4B, cAMP specific | |
| *Pde4dip* | H | phosphodiesterase 4D interacting protein | |
| *Pdk2* | L | pyruvate dehydrogenase kinase, isozyme 2 | |
| *Pdlim3* | H | PDZ and LIM domain 3 | |
| *Pfkfb1* | H | 6-phosphofructo-2-kinase/fructose-2,6-biphosphatase 1 | |
| *Pfkfb4* | L | 6-phosphofructo-2-kinase/fructose-2,6-biphosphatase 4 | |
| *Pgam2* | H | phosphoglycerate mutase 2 (muscle) | |
| *Pglyrp1* | L | peptidoglycan recognition protein 1 | |
| *Pgm1* | H | phosphoglucomutase 1 | |
| *Pigq* | L | phosphatidylinositol glycan anchor biosynthesis, class Q | |
| *Pik3ip1* | L | phosphoinositide-3-kinase interacting protein 1 | |
| *Pip5k1b* | L | phosphatidylinositol-4-phosphate 5-kinase, type I, beta | |
| *Pkia* | H | protein kinase (cAMP-dependent, catalytic) inhibitor alpha | |
| *Pkm2* | H | pyruvate kinase, muscle | |
| *Pla2g2d* | L | phospholipase A2, group IID | |
| *Pla2g7* | L | phospholipase A2, group VII (platelet-activating factor acetylhydrolase, plasma) | |
| *Plag1* | L | pleiomorphic adenoma gene 1 | |
| *Plcd4* | H | phospholipase C, delta 4 | |
| *Plekhb1* | L | pleckstrin homology domain containing, family B (evectins) member 1 | |
| *Plp1* | L | proteolipid protein 1 | |
| *Polr1b* | L | polymerase (RNA) I polypeptide B | |
| *Ppm2c* | L | protein phosphatase 2C, magnesium dependent, catalytic subunit | |
| *Ppp1r1a* | H | protein phosphatase 1, regulatory (inhibitor) subunit 1A | |
| *Ppp1r3c* | H | protein phosphatase 1, regulatory (inhibitor) subunit 3C | |
| *Ppp3cb* | L | protein phosphatase 3, catalytic subunit, beta isoform | |
| *Prdx1* | L | peroxiredoxin 1 | |
| *Prdx3* | L | peroxiredoxin 3 | |
| *Prelp* | L | proline arginine-rich end leucine-rich repeat protein | |
| *Prg2* | L | proteoglycan 2, bone marrow | |
| *Prim1* | L | DNA primase, p49 subunit | |
| *Prkaa2* | H | protein kinase, AMP-activated, alpha 2 catalytic subunit | |
| *Prkab2* | L | protein kinase, AMP-activated, beta 2 non-catalytic subunit | |
| Supplementary Table 1 continued | | | |
| **Matrix Formation (down) Clusters continued** | | | |
| **Gene Symbol** | **Cluster** | **Gene Name** | |
| *Prkcb* | L | protein kinase C, beta | |
| *Prkg2* | L | protein kinase, cGMP-dependent, type II | |
| *Prom1* | L | prominin 1 | |
| *Ptgds2* | L | prostaglandin D2 synthase 2, hematopoietic | |
| *Ptk2b* | L | PTK2B protein tyrosine kinase 2 beta | |
| *Ptp4a1* | L | protein tyrosine phosphatase 4a1 | |
| *Ptpn18* | L | protein tyrosine phosphatase, non-receptor type 18 | |
| *Ptpn6* | L | protein tyrosine phosphatase, non-receptor type 6 | |
| *Ptprc* | L | protein tyrosine phosphatase, receptor type, C | |
| *Pttg1* | L | pituitary tumor-transforming 1 | |
| *Pvalb* | H | parvalbumin | |
| *Pygl* | L | phosphorylase, glycogen, liver | |
| *Rac2* | L | ras-related C3 botulinum toxin substrate 2 (rho family, small GTP binding protein Rac2) | |
| *Rbp1* | H | retinol binding protein 1, cellular | |
| *Reln* | L | reelin | |
| *Retnlg* | L | resistin-like gamma | |
| *RGD1305288* | L | similar to chromosome 14 open reading frame 94 | |
| *RGD1307279* | L | similar to RIKEN cDNA 2700002I20 | |
| *RGD1307935* | L | similar to Hypothetical protein MGC18716 | |
| *RGD1308734* | L | similar to RIKEN cDNA 1100001H23 | |
| *RGD1309522* | L | similar to hypothetical protein FLJ22624 | |
| *RGD1309676* | L | similar to RIKEN cDNA 5730469M10 | |
| *RGD1359600* | L | LEA_4 domain containing protein RGD1359600 | |
| *RGD1565355* | L | similar to fatty acid translocase/CD36 | |
| *Rhd* | L | Rh blood group, D antigen | |
| *Rpl15* | L | ribosomal protein L15 | |
| *Rsad2* | L | radical S-adenosyl methionine domain containing 2 | |
| *Rtn2* | L | reticulon 2 | |
| *Samsn1* | L | SAM domain, SH3 domain and nuclear localization signals, 1 | |
| *Sclt1* | L | sodium channel and clathrin linker 1 | |
| *Scn4a* | H | sodium channel, voltage-gated, type 4, alpha subunit | |
| *Scn4b* | H | sodium channel, type IV, beta | |
| *Scn7a* | L | sodium channel, voltage-gated, type VII, alpha | |
| *Sdpr* | L | serum deprivation response | |
| *Sell* | L | selectin, lymphocyte | |
| *Selplg* | L | selectin, platelet (p-selectin) ligand | |
| *Sema6c* | L | sema domain, transmembrane domain (TM), and cytoplasmic domain, (semaphorin) 6C | |
| *Serpinb2* | L | serine (or cysteine) peptidase inhibitor, clade B, member 2 | |
| *Sfrp4* | H | secreted frizzled-related protein 4 | |
|  | | | |
| Supplementary Table 1 continued | | | |
| **Matrix Formation (down) Clusters continued** | | | |
| **Gene Symbol** | **Cluster** | **Gene Name** | |
| *Sgtb* | L | small glutamine-rich tetratricopeptide repeat (TPR)-containing, beta | |
| *Siah1a* | L | seven in absentia 1A | |
| *Sla* | L | src-like adaptor | |
| *Slc14a1* | L | solute carrier family 14 (urea transporter), member 1 | |
| *Slc15a2* | L | solute carrier family 15 (H+/peptide transporter), member 2 | |
| *Slc16a1* | L | solute carrier family 16, member 1 (monocarboxylic acid transporter 1) | |
| *Slc16a3* | H | solute carrier family 16, member 3 (monocarboxylic acid transporter 4) | |
| *Slc16a6* | L | solute carrier family 16, member 6 (monocarboxylic acid transporter 7) | |
| *Slc22a3* | H | solute carrier family 22 (extraneuronal monoamine transporter), member 3 | |
| *Slc25a11* | L | solute carrier family 25 (mitochondrial carrier; oxoglutarate carrier), member 11 | |
| *Slc25a30* | L | solute carrier family 25, member 30 | |
| *Slc2a1* | H | solute carrier family 2 (facilitated glucose transporter), member 1 | |
| *Slc38a1* | L | solute carrier family 38, member 1 | |
| *Slc38a3* | L | solute carrier family 38, member 3 | |
| *Slc4a1* | L | solute carrier family 4 (anion exchanger), member 1 | |
| *Slc6a20* | L | solute carrier family 6 (neurotransmitter transporter), member 20 | |
| *Slc6a4* | L | solute carrier family 6 (neurotransmitter transporter, serotonin), member 4 | |
| *Slco1a4* | H | solute carrier organic anion transporter family, member 1a4 | |
| *Slfn3* | L | schlafen 3 | |
| *Smpx* | H | small muscle protein, X-linked | |
| *Snca* | L | synuclein, alpha (non A4 component of amyloid precursor) | |
| *Sost* | L | sclerosteosis | |
| *Speg* | L | SPEG complex locus | |
| *Spon1* | L | spondin 1 | |
| *Spta1* | L | spectrin, alpha, erythrocytic 1 (elliptocytosis 2) | |
| *Srgn* | L | serglycin | |
| *Srpk3* | H | SFRS protein kinase 3 | |
| *St8sia5* | H | ST8 alpha-N-acetyl-neuraminide alpha-2,8-sialyltransferase 5 | |
| *Stap1* | L | signal transducing adaptor family member 1 | |
| *Stc2* | L | stanniocalcin 2 | |
| *Stom* | L | stomatin | |
| *Strbp* | L | spermatid perinuclear RNA binding protein | |
| *Stx7* | L | syntaxin 7 | |
| Supplementary Table 1 continued | | | |
| **Matrix Formation (down) Clusters continued** | | | |
| **Gene Symbol** | **Cluster** | **Gene Name** | |
| *Sub1* | L | SUB1 homolog (S. cerevisiae) | |
| *Sult1a1* | H | sulfotransferase family, cytosolic, 1A, phenol-preferring, member 1 | |
| *Sv2b* | L | synaptic vesicle glycoprotein 2b | |
| *Syne1* | L | spectrin repeat containing, nuclear envelope 1 | |
| *Synm* | H | synemin | |
| *Tacc3* | L | transforming, acidic coiled-coil containing protein 3 | |
| *Tas2r119* | L | taste receptor, type 2, member 119 | |
| *Tc2n* | L | tandem C2 domains, nuclear | |
| *Tceb1* | L | transcription elongation factor B (SIII), polypeptide 1 | |
| *Tfpi* | L | tissue factor pathway inhibitor (lipoprotein-associated coagulation inhibitor) | |
| *Tgfbr3* | L | transforming growth factor, beta receptor III | |
| *Tmem38b* | H | transmembrane protein 38B | |
| *Tmod1* | H | tropomodulin 1 | |
| *Tmpo* | L | thymopoietin | |
| *Tnfrsf14* | L | tumor necrosis factor receptor superfamily, member 14 | |
| *Tnfsf10* | H | tumor necrosis factor (ligand) superfamily, member 10 (TRAIL) | |
| *Tnni2* | H | troponin I type 2 | |
| *Tnnt3* | H | troponin T type 3 | |
| *Top2a* | L | topoisomerase (DNA) II alpha | |
| *Tpm2* | L | tropomyosin 2 | |
| *Tpmt* | L | thiopurine S-methyltransferase | |
| *Trdn* | H | triadin | |
| *Trim54* | H | tripartite motif-containing 54 | |
| *Trim55* | H | tripartite motif-containing 55 | |
| *Tsc22d3* | H | TSC22 domain family, member 3 | |
| *Tspan5* | L | tetraspanin 5 | |
| *Tspan8* | H | tetraspanin 8 | |
| *Tuba8* | H | tubulin, alpha 8 | |
| *Txnip* | L | thioredoxin interacting protein | |
| *Tyms* | L | thymidylate synthetase | |
| *Ubac1* | H | UBA domain containing 1 | |
| *Ucp2* | L | uncoupling protein 2 (mitochondrial, proton carrier) | |
| *Uros* | L | uroporphyrinogen III synthase | |
| *Usp1* | L | ubiquitin specific peptidase 1 | |
| *Usp15* | L | ubiquitin specific peptidase 15 | |
| *Vamp1* | L | vesicle-associated membrane protein 1 | |
| *Vav1* | L | vav 1 guanine nucleotide exchange factor | |
| *Vcam1* | L | vascular cell adhesion molecule 1 | |
| *Vezt* | L | vezatin, adherens junctions transmembrane protein | |
| *Vof16* | L | ischemia related factor vof-16 | |
| Supplementary Table 1 continued | | | |
| **Matrix Formation (down) Clusters continued** | | | |
| **Gene Symbol** | **Cluster** | **Gene Name** | |
| *Vrk1* | L | vaccinia related kinase 1 | |
| *Xirp2* | H | xin actin-binding repeat containing 2 | |
| *Xpo1* | L | exportin 1, CRM1 homolog (yeast) | |
| *Zbtb16* | H | zinc finger and BTB domain containing 16 | |
| *Znf622* | H | zinc finger protein 622 | |
